# Supplementary material for: Spatial triple-correlation spectroscopy reveals heterotrimer dynamics in live cells
Source: Biophys J. 2026 Mar 5;125(7):1723–36. doi: 10.1016/j.bpj.2026.03.007 (PMC13351891; doi:10.1016/j.bpj.2026.03.007)
Supplement: Document S1. Figures S1–S12 [file mmc1.pdf]

Biophysical Journal, Volume *125*

Supplemental information

Spatial triple-correlation spectroscopy reveals heterotrimer dynamics in live cells

Julissa Sanchez-Velasquez, Tao Sun, Xiaomeng Zhang, and Elizabeth Hinde

Supplementary information

Spatial Triple-Correlation Spectroscopy (S3CS) Reveals Heterotrimer Dynamics in Live Cells

Julissa Sanchez-Velasquez[1], Tao Sun[1], Xiaomeng Zhang[1], Elizabeth Hinde[1*].

[1]School of Physics, University of Melbourne, Melbourne, Victoria Australia.

* Corresponding author: elizabeth.hinde@unimelb.edu.au

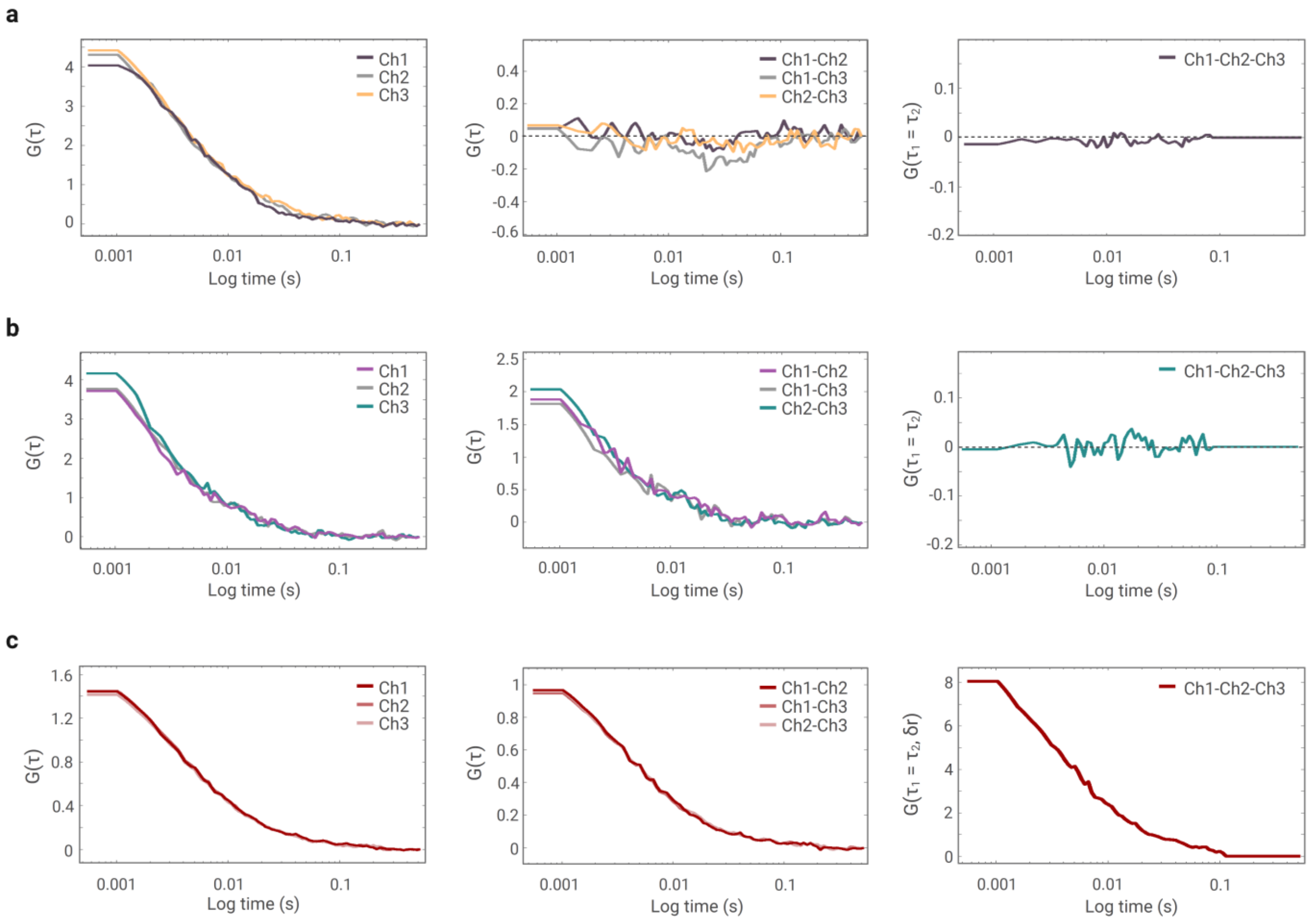

Supplementary Fig. 1. Positive S3CS triple-correlation amplitudes are specific to heterotrimeric assemblies. a-c, Representative autocorrelation (G_1, G_2, G_3) (left), cross-correlation ($G_{1\text{-}2}$, $G_{1\text{-}3}$, $G_{2\text{-}3}$) (middle), and triple correlation ($\tau_1 = \tau_2$) (right) functions obtained from simulations considering a homogeneous population of monomers (**a**), dimers (**b**), and heterotrimers (**c**) undergoing isotropic diffusion ($N = 200$, $D = 10\ \mu m^2\ s^{-1}$).

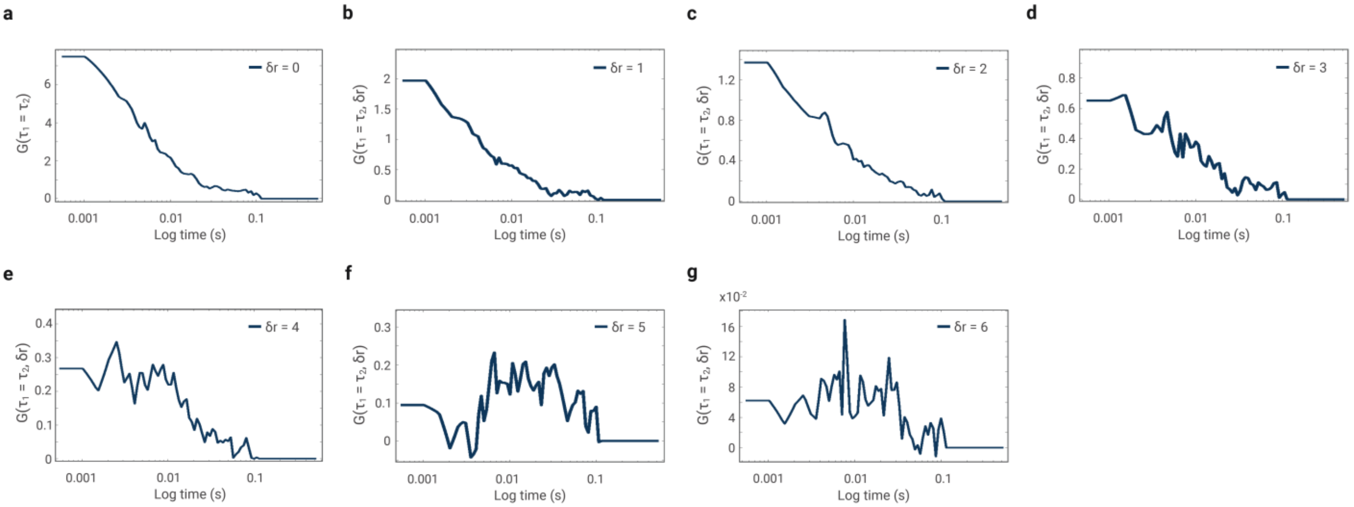

Supplementary Fig. 2. S3CS across increasing spatial offsets. a, $G_{123}(\tau_1 = \tau_2)$ profile obtained from a simulation considering a homogeneous population of heterotrimers undergoing isotropic diffusion (N = 200, D = 10 $\mu m^2\ s^{-1}$). **b-g,** $G_{123}(\tau_1 = \tau_2, \delta r)$ profiles computed at increasing spatial offsets (δr = 1, 2, 3, 4, 5, 6) from the simulation in **a**.

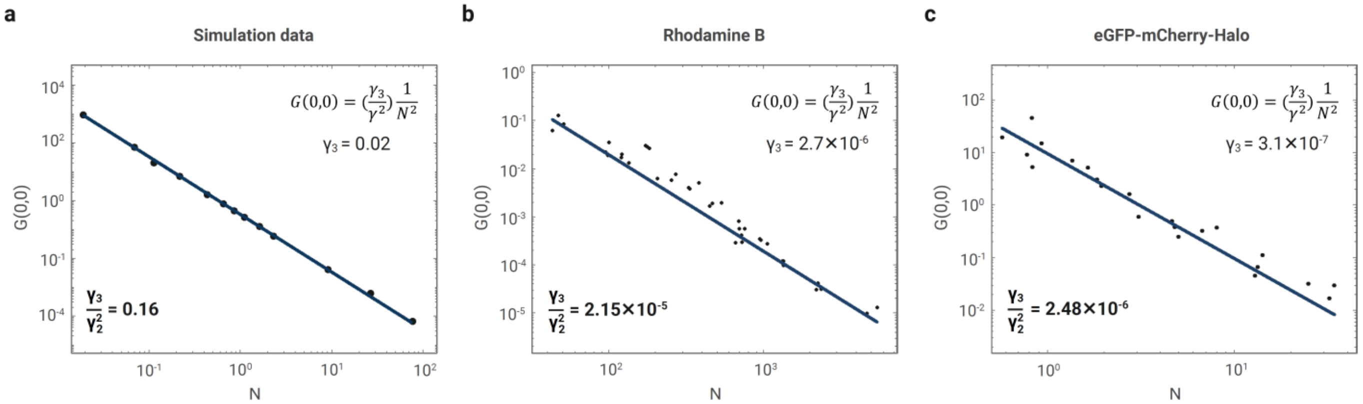

Supplementary Fig. 3. Determining the correction factor γ_3 for triple-correlation analysis. a-c, To account for the intensity distribution within the triple-correlation observation volume, triple-correlation amplitudes, $G_{123}(0,0)$, were measured across varying concentrations of simulated heterotrimers (**a**), Rhodamine B (**b**), and the eGFP-mCherry-Halo-JF646 construct (**c**). γ_3 was obtained by fitting $G_{123}(0,0)$ versus the apparent number of molecules (N) to the equation shown in the log-log plots. The resulting γ_3 values were used to calculate the ratio of triple- to double-correlation focal volumes, γ_3/γ, with γ fixed to $1/\sqrt{8}$, the value predicted for a Gaussian focal volume.

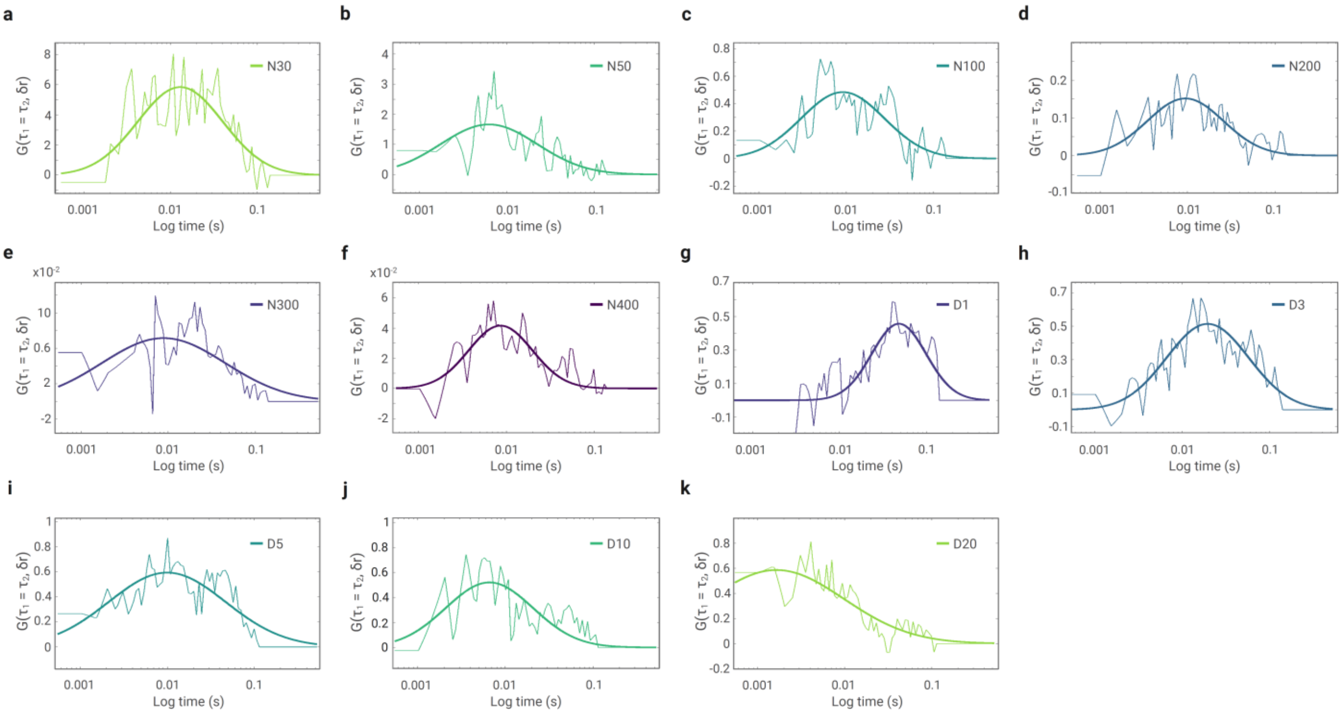

Supplementary Fig. 4. Underlying S3CS correlation profiles corresponding to Fig. 2n,p. a-f, $G_{123}(\tau_1 = \tau_2, \delta r = 6)$ profiles obtained from simulations considering a homogeneous population of heterotrimers undergoing isotropic diffusion ($D = 10\ \mu m^2\ s^{-1}$) at increasing particle numbers (N = 30 - 400). **g-k,** $G_{123}(\tau_1 = \tau_2, \delta r = 6)$ profiles obtained from simulations considering a homogeneous population of heterotrimers with fixed particle number (N = 100) across increasing diffusion coefficients ($D = 1 – 20\ \mu m^2\ s^{-1}$). Solid curves indicate fits obtained using a general Gaussian model.

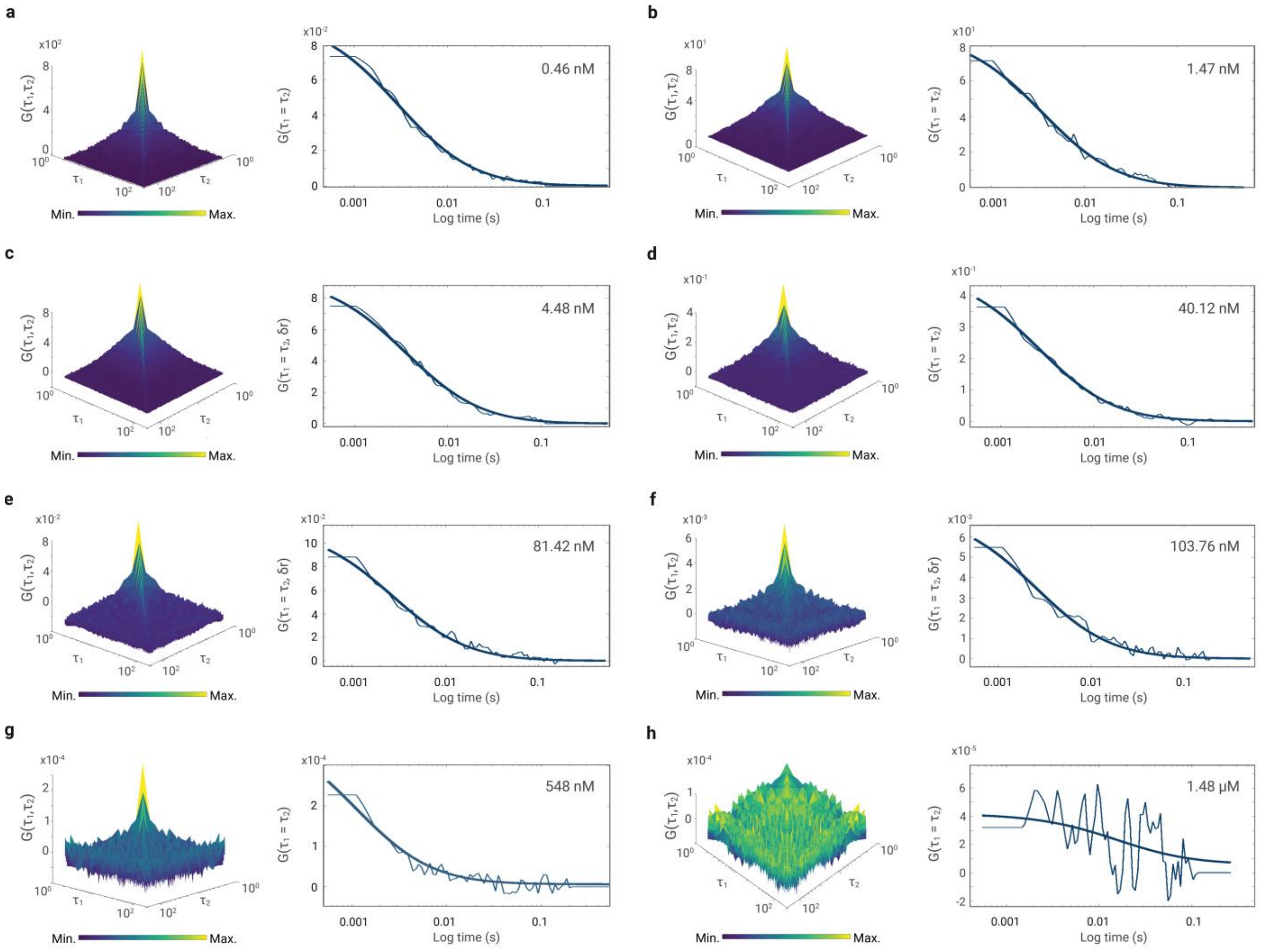

Supplementary Fig. 5. Concentration dependence of S3CS correlation profiles. a-h, Representative averaged S3CS correlation surfaces, $G_{123}(\tau_1, \tau_2)$, obtained from simulations considering a homogeneous population of heterotrimers undergoing isotropic diffusion (D = 10 μm^2 s^{-1}) across increasing particle concentrations within the observation volume (0.46 nM – 1.48 μM). For each condition, the $\tau_1 = \tau_2$ diagonal extracted from the 2D triple correlation surface is shown on the right. Solid lines indicate fits using a one-component diffusion model assuming a 3D Gaussian point spread function (PSF).

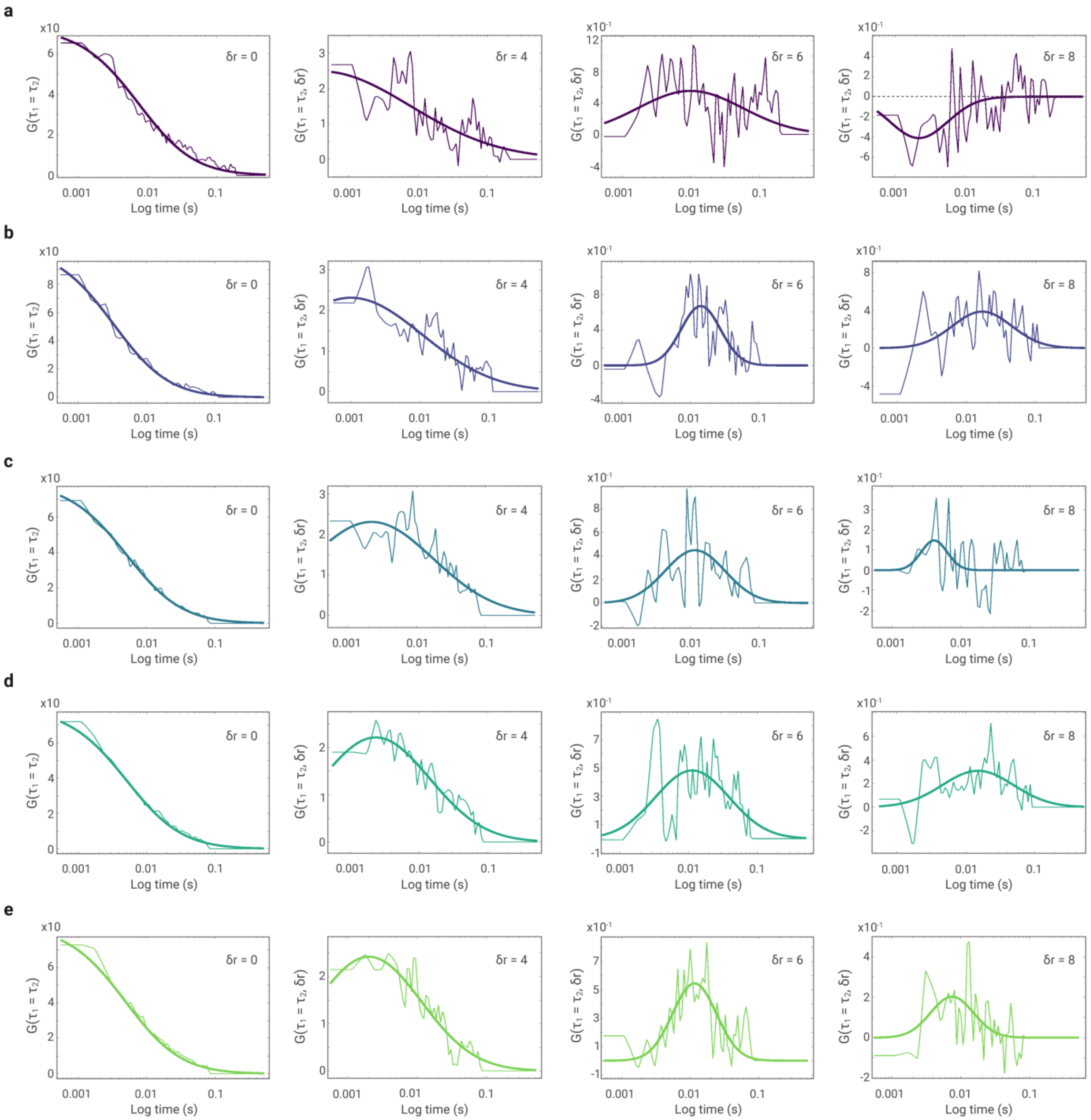

Supplementary Fig. 6. Effect of increasing statistical sampling in S3CS analysis. a-e, $G_{123}(\tau_1 = \tau_2, \delta r)$ profiles computed at increasing spatial offsets (δr = 0, 4, 6, and 8) obtained from simulations considering a homogeneous population of heterotrimers undergoing isotropic diffusion (N = 100, D = 10 $\mu m^2\ s^{-1}$). S3CS analyses were calculated using progressively increased statistical sampling, corresponding to 100 000 (**a**), 200 000 (**b**), 300 000 (**c**), 400 000 (**d**), and 500 000 (**e**) scanned lines. Solid lines indicate fits obtained using a one-component diffusion model assuming a 3D Gaussian point spread function (PSF) for δr = 0, or a general Gaussian model for δr > 0. Increasing sampling improves S3CS profiles' stability and smoothness, particularly at larger spatial offsets.

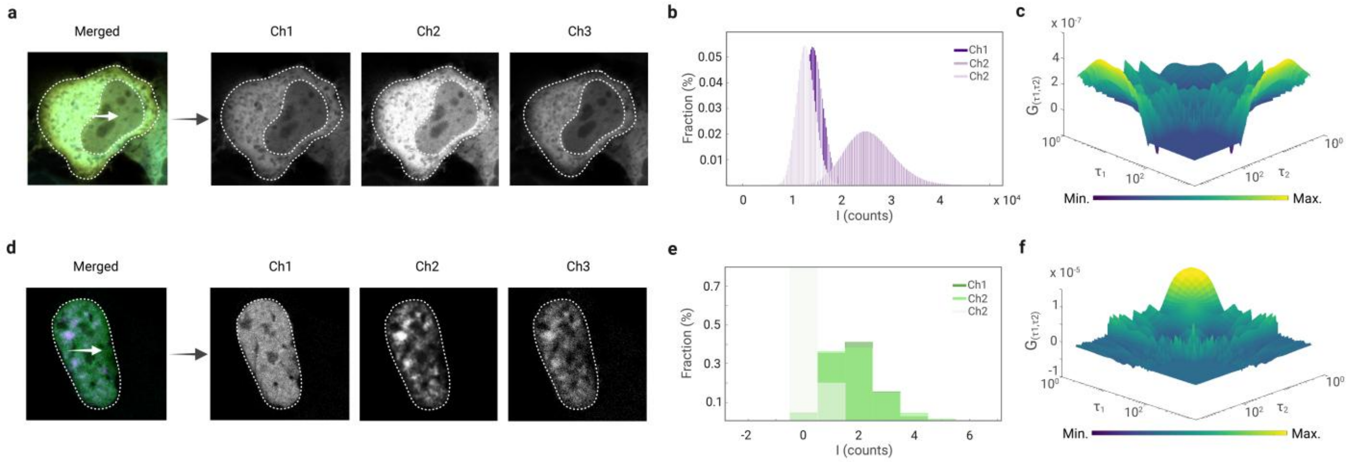

Supplementary Fig. 7. Impact of protein concentration on S3CS in live cells. a, Representative confocal image of a HeLa cell transfected with the eGFP-mCherry-Halo-JF646 construct. A three-color merged confocal image is shown on the left. Confocal images for the eGFP (Ch1), mCh (Ch2), and Halo-JF646 (Ch3) channels are shown on the right. The line across the nuclear envelope positions the directionality of the three-channel line scan. **b,** Intensity histograms for Ch1, Ch2, and Ch3 from the cell presented in **a**. **c,** $G_{123}(\tau_1, \tau_2)$ profile for the cell shown in **a**. At high protein concentration within the observation volume (~33 μM), the correlation profile exhibits increased noise. **d,** Representative confocal image of a HeLa cell co-transfected with eGFP-NF-YB, mCherry-NF-YA, and NF-YC-Halo646. A three-color merged confocal image is shown on the left. Confocal images for the eGFP (Ch1), mCh (Ch2), and Halo-JF646 (Ch3) channels are shown on the right. The line inside the nucleus positions the directionality of the three-channel line scan. **e,** Intensity histograms for Ch1, Ch2, and Ch3 from the cell presented in **d**. **f,** $G_{123}(\tau_1, \tau_2)$ profile for the cell shown in **d**. At low protein concentration (< 2 μM), a clear correlation peak is observed. I, intensity.

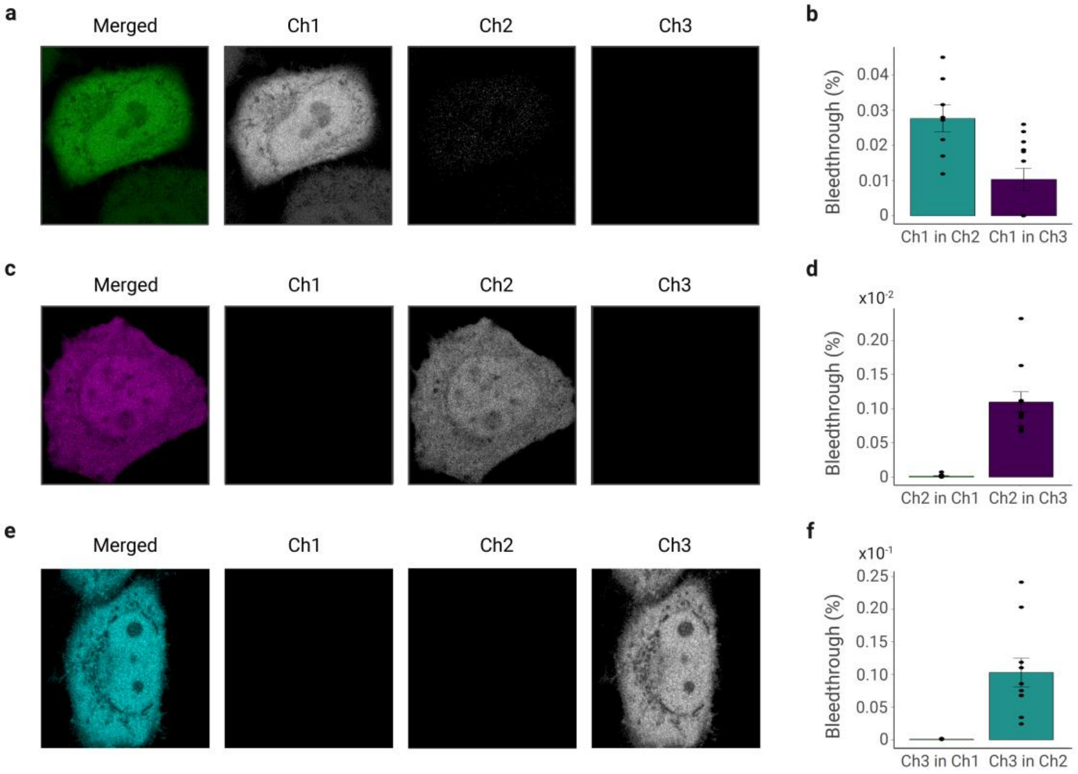

Supplementary Fig. 8. Quantitative validation of spectral bleedthrough in three-color confocal acquisitions. a, Representative confocal image of a HeLa cell expressing eGFP alone. Left: three-color merged image. Right: single-channel images for Ch1 (eGFP detection), Ch2, and Ch3. **b,** Quantification of spectral bleedthrough from eGFP into Ch2 and Ch3. For each cell, fluorescence intensity measured in Ch1 was normalized to 100%, and the corresponding signal detected in Ch2 or Ch3 was expressed as a percentage of the Ch1 intensity. **c,** Representative confocal image of a HeLa cell expressing mCherry alone, shown as a merged image (left) and individual detection channels (Ch1, Ch2, Ch3, right). **d,** Quantification of spectral bleedthrough from mCherry into Ch1 and Ch3, calculated by normalizing Ch2 (mCherry channel) intensity to 100% and expressing signals detected in the other channels as a percentage of the Ch2 intensity. **e,** Representative confocal images of a HeLa cell expressing Halo-JF646 alone, shown as merged and single-channel images. **f,** Quantification of spectral bleedthrough from Halo-JF646 into Ch1 and Ch2, with Ch3 (Halo-JF646 channel) normalized to 100% and residual signal in Ch1 and Ch2 expressed as a percentage of Ch3 intensity. In **b**,**d**,**f** bars represent mean ± s.e.m. Across all single-color controls, bleedthrough into non-corresponding detection channels was minimal, confirming effective spectral separation and validating the suitability of this imaging configuration for S3CS analyses.

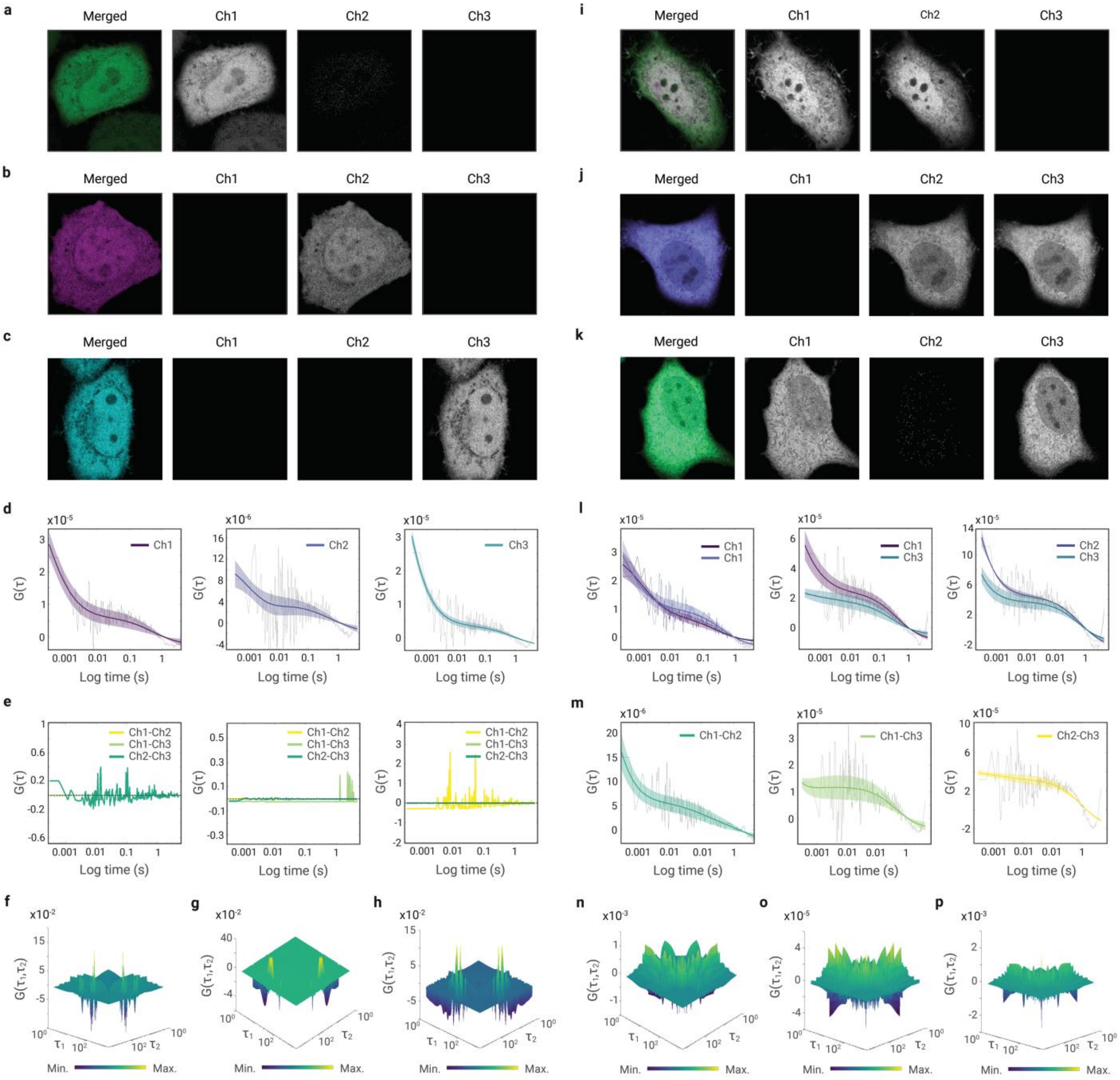

Supplementary Fig. 9. Validation of bleedthrough suppression and specificity in S3CS using independent fluorophores and dual-color constructs. a-c, Representative confocal images of HeLa cells expressing eGFP (**a**), mCherry (**b**), or Halo-JF646 (**c**). Three-color merged confocal images are shown on the left, with corresponding single-channel images for eGFP (Ch1), mCh (Ch2), and Halo-JF646 (Ch3) shown on the right. **d,** Autocorrelation functions (ACF) computed from the corresponding detection channel expressing eGFP (left), mCherry (center), and Halo-JF646 (right). **e,** Cross correlation functions (CCF) computed between all possible channel pairs for cells expressing single fluorescent proteins: eGFP (left), mCherry (center), and Halo-JF646 (right). **f-h,** Representative $G_{123}(\tau_1, \tau_2)$ profiles obtained from cells expressing eGFP (**f**), mCherry (**g**), or Halo-JF646 (**h**). **i-k,** Representative confocal images of HeLa

cells expressing dual-color fusion constructs: mCherry-eGFP (**i**), eGFP-Halo-JF646 (**j**), or mCherry-Halo-JF646 (**k**). Three-color merged images are shown on the left, with individual detection channels shown on the right. **l,** ACFs computed from the corresponding fluorescence channels for each dual-color construct: mCherry-eGFP (left), eGFP-Halo-JF646 (center), and mCherry-Halo-JF646 (right). **m,** CCFs computed between the two fluorophore channels present in each dual-color construct, mCherry-eGFP (left), eGFP-Halo-JF646 (center), and mCherry-Halo-JF646 (right). **n-p,** Representative $G_{123}(\tau_1, \tau_2)$ profiles from cells expressing mCherry-eGFP (**n**), eGFP-Halo-JF646 (**o**), and mCherry-Halo-JF646 (**p**). In all cases, triple-correlation profiles were absent, confirming negligible spectral bleedthrough and high specificity of the S3CS measurement.

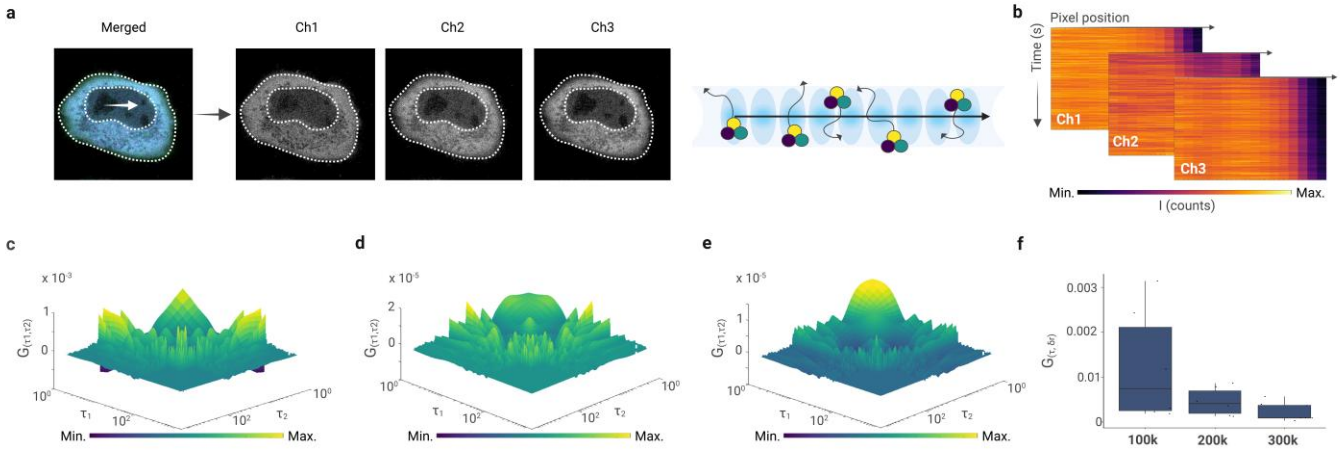

Supplementary Fig. 10. Improved definition of S3CS profiles in live cells with increased line-scan statistics. a, Representative confocal image of a HeLa cell transfected with the eGFP-mCherry-Halo-JF646 triple construct. A three-color merged confocal image is shown on the left. Confocal images for the eGFP (Ch1), mCh (Ch2), and Halo-JF646 (Ch3) channels are shown on the right. The line inside the nucleus positions the directionality of the three-channel line scan. **b,** The fluctuations in fluorescence intensity that result from eGFP-mCherry-Halo-JF646 diffusing in and out of pixels along the line scan are recorded and plotted in intensity carpet representations, where the x-axis is pixel position, and the y-axis is time. **c-e,** $G_{123}(\tau_1, \tau_2)$ profiles from line scans performed using 100 000 (**c**), 200 000 (**d**), and 300 000 (**e**) lines, demonstrating improved correlation peak definition with increased scan statistics. In **c** and **d**, the $G_{123}(\tau_1, \tau_2)$ profiles were obtained from HeLa cells transfected with the eGFP-mCherry-Halo-JF646 triple construct; in **e**, the $G_{123}(\tau_1, \tau_2)$ profile was obtained from a HeLa cell co-transfected with eGFP-NF-YB, mCherry-NF-YA, and NF-YC-Halo646. **f,** Boxplots summarizing the maximum $G_{123}(\tau_1 = \tau_2)$ amplitudes, illustrating reduced variability and improved correlation signal robustness as the number of lines increases from 100 000 (100k) to 300 000 (300k). Each box plot shows the median (middle line) and interquartile range (boxes). The bottom and top of each box indicate the 25th and 75th percentiles, respectively, while whiskers represent the minimum and maximum.

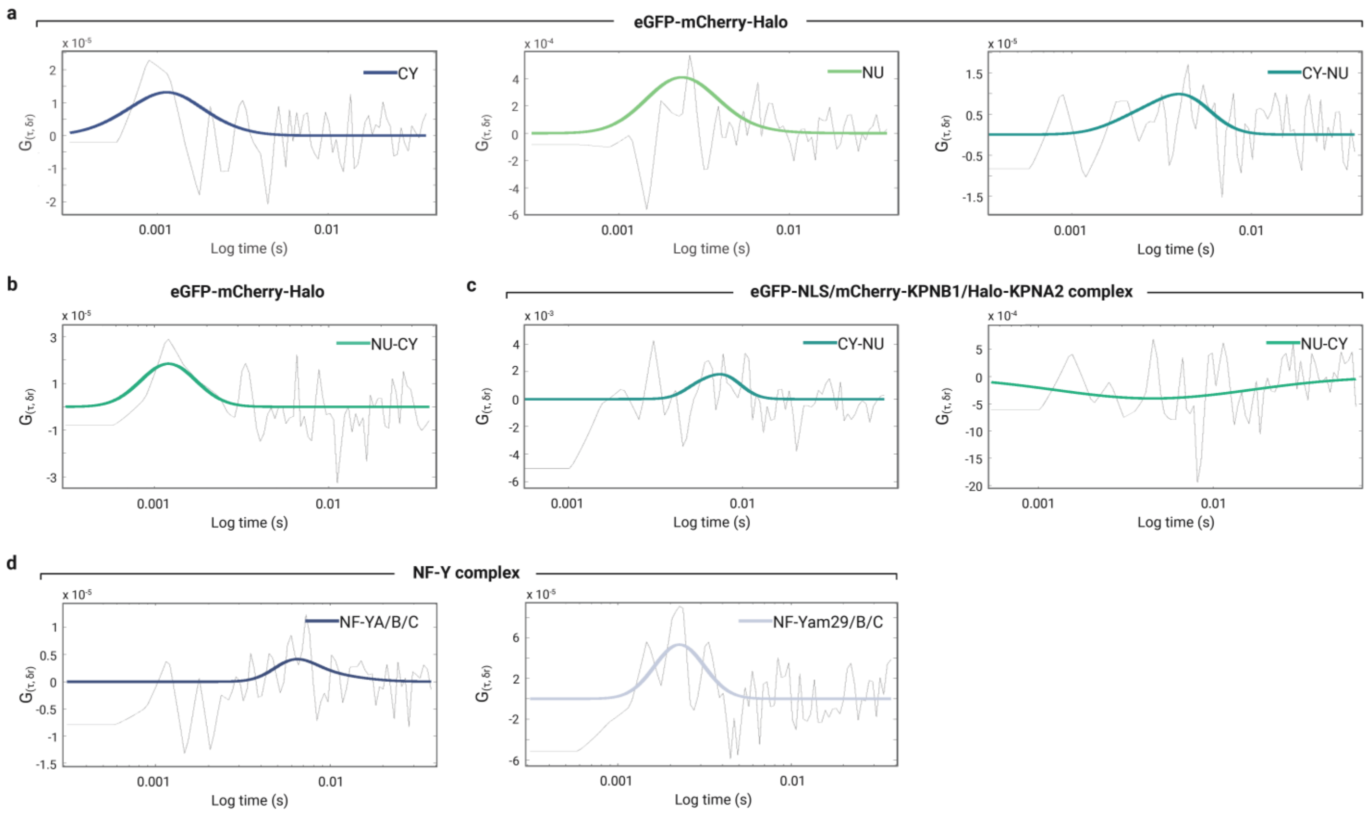

Supplementary Fig. 11. Underlying raw data for the fitted S3CS profiles shown in Figs. 4-6. a,b, Average $G_{123}(\tau_1 = \tau_2, \delta r = 3)$ profile for eGFP-mCherry-Halo-JF646 mobility within the cytoplasm (CY), nucleus (NU), and across the nuclear envelope in the cytoplasm to nucleus direction (**a**) and in the nucleus to cytoplasm direction (**b**). The solid thick lines represent the fitted curves (reference Fig. 4). **c,** Average $G_{123}(\tau_1 = \tau_2, \delta r = 3)$ profile for eGFP-NLS/mCherry-KPNB1/Halo-JF646-KPNA2 complex mobility across the nuclear envelope in the cytoplasm to nucleus direction (left) and in the nucleus to cytoplasm direction (right). The solid thick lines represent the fitted curves (reference Fig. 5). **d,** Average $G_{123}(\tau_1 = \tau_2, \delta r = 3)$ profiles for NF-YA/B/C (left) and NF-Yam29/B/C (right) complexes mobility inside the nucleus. The solid thick lines represent the fitted curves (reference Fig. 6).

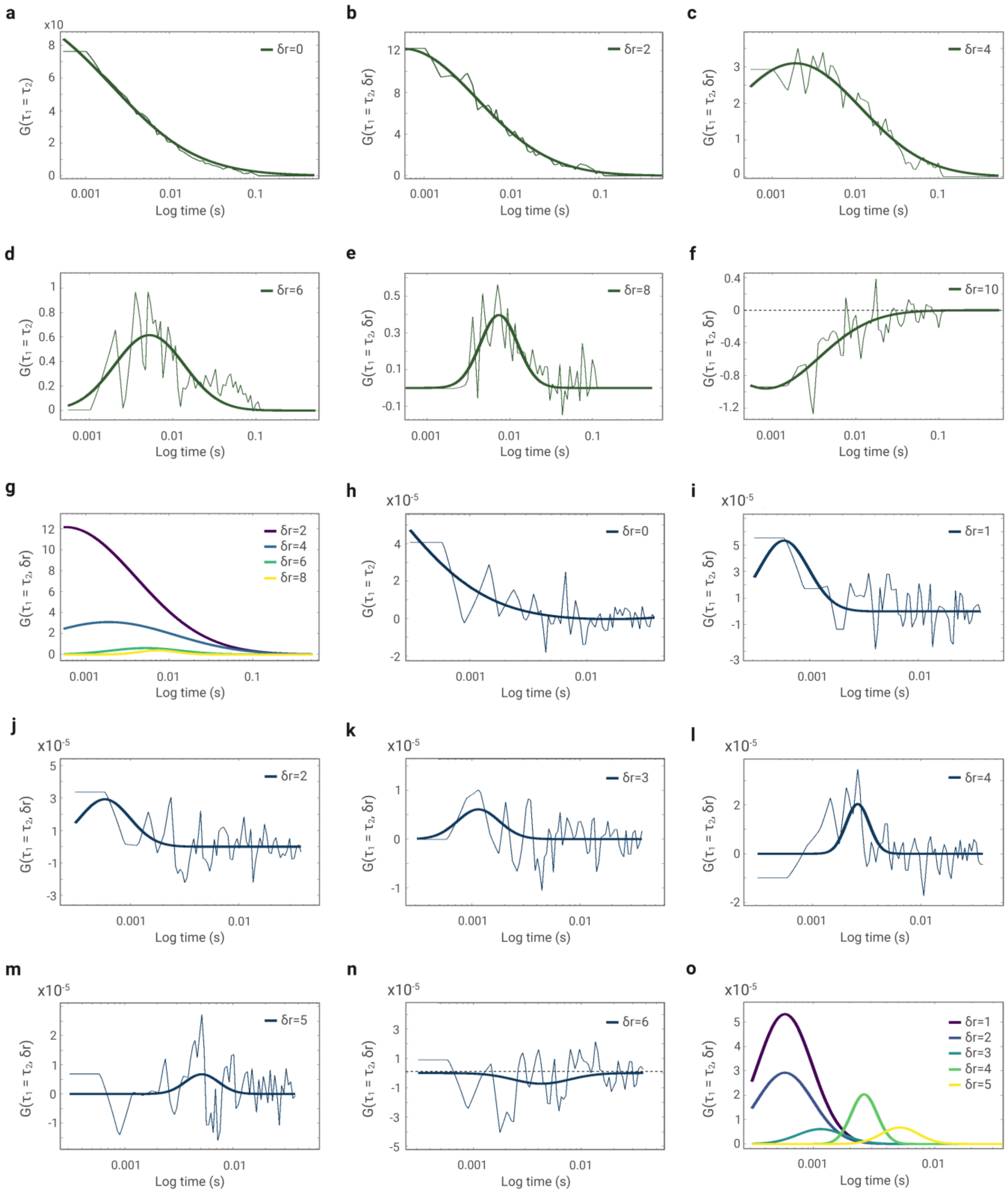

Supplementary Fig. 12. Spatial range of detectable S3CS correlations in simulations and live cells. **a,** Schematic of S3CS analysis, $G_{123}(\tau_1 = \tau_2)$, from simulations considering a homogeneous population of heterotrimers undergoing isotropic diffusion (N = 100, D = 10 μm^2 s^{-1}). **b-f,** Simulated $G_{123}(\tau_1 = \tau_2, \delta r)$

profiles computed at increasing spatial offsets (δr = 2, 4, 6, 8, 10) from the simulation in **a**, showing well-defined correlation profiles at small-to-intermediate offsets, with progressive signal attenuation and loss of detectable correlation at the largest offset (δr = 10). **g,** Fitted $G_{123}(\tau_1 = \tau_2, \delta r)$ profiles corresponding to panels **b-e**. **h,** Experimental $G_{123}(\tau_1 = \tau_2)$ measured in live HeLa cells expressing the eGFP-mCherry-Halo646 triple-labelled construct. **i-n,** Experimental $G_{123}(\tau_1 = \tau_2, \delta r)$ profiles computed at increasing spatial offsets (δr = 1, 2, 3, 4, 5, 6), showing a progressive reduction in correlation amplitude and increased noise at larger offsets. **o,** Fitted $G_{123}(\tau_1 = \tau_2, \delta r)$ profiles corresponding to panels **i-m**. Solid lines indicate fits obtained using a one-component diffusion model assuming a 3D Gaussian point spread function (PSF) for $\delta r = 0$, or a general Gaussian model for $\delta r > 0$.
