# Supplementary material for: Spatial triple-correlation spectroscopy reveals heterotrimer dynamics in live cells
Source: Biophys J. 2026 Mar 5;125(7):1723–36. doi: 10.1016/j.bpj.2026.03.007 (PMC13351891; doi:10.1016/j.bpj.2026.03.007)
Supplement: Document S2. Article plus supporting material [file mmc2.pdf]

Biophysical Journal
Article

Spatial triple-correlation spectroscopy reveals heterotrimer dynamics in live cells

Julissa Sanchez-Velasquez,[1] Tao Sun,[1] Xiaomeng Zhang,[1] and Elizabeth Hinde[1,*]
[1]School of Physics, University of Melbourne, Melbourne, Victoria, Australia

ABSTRACT Heterotrimeric protein complexes are central regulators of intracellular signaling, yet their dynamic assembly and transport in living cells remain difficult to resolve. Here, we present spatial triple-correlation spectroscopy (S3CS), a fluorescence fluctuation method that integrates three-channel line scan microscopy with a spatial triple-correlation function to directly detect fluorescent heterotrimers and map their movement relative to subcellular architecture. Simulations establish that S3CS quantitatively captures heterotrimer formation, local diffusion, and long-range transport, while live-cell experiments confirm its specificity for following fluorescent ternary assemblies in the presence of free independent subunits. Applying S3CS to the importin-α/importin-β/NLS cargo complex revealed directional, irreversible nuclear import, whereas analysis of the NF-Y transcription factor showed that heterotrimer assembly precedes chromatin engagement. By selectively resolving the dynamics of fluorescent ternary assemblies at intracellular boundaries, S3CS provides a versatile platform to dissect how heterotrimeric signaling complexes employ molecular interactions to navigate the dynamic structural framework of the living cell.

SIGNIFICANCE Cells rely on multi-protein complexes to orchestrate essential functions, yet monitoring three-part assemblies in living cells has remained challenging. Here we introduce spatial triple-correlation spectroscopy (S3CS), a method that directly visualizes and quantifies heterotrimer formation, dynamics, and directional transport with subcellular precision. S3CS selectively detects heterotrimers, tracks local to long-range diffusion, and maps transport across barriers such as the nuclear envelope. Applied to biologically important complexes, including canonical nuclear import machinery, it reveals real-time assembly dynamics and functional consequences of subunit interactions. By providing a generalizable tool for probing complex protein behavior in living cells, S3CS opens new avenues for understanding how protein networks regulate cellular organization and signaling.

INTRODUCTION

Access to microscopy methods capable of spatiotemporally tracking protein transport during homotypic and heterotypic interactions, within the crowded and compartmentalized environment of a living cell, is essential for decoding the molecular mechanisms that drive intracellular signaling. This is because protein transport is primarily governed by diffusion, a default mode of motion that is dynamically reshaped by molecular events such as changes in hydrodynamic radius, molecular accessibility, or binding affinity.[1–6] As a result, both self-association and heterotypic complex formation can redirect a protein's diffusive trajectory, ultimately determining where, when, and how it performs its biological function. Recent advances in protein-specific fluorescent labeling,[7,8] combined with multicolor adaptations of microscopy methods, such as single-particle tracking[9–11] and fluorescence correlation spectroscopy (FCS),[12–15] have significantly improved our ability to detect protein interactions during intracellular transport. However, one stoichiometric configuration that remains particularly elusive is the heterotrimer. Despite its central role in signal transduction pathways, from the plasma membrane to the cell nucleus, no microscopy method exists that can track heterotrimer movement across the intracellular landscape. A promising starting point may be triple FCS (F3CS),[16–18] given its proven sensitivity to heterotrimer formation. Yet, to fully resolve how these complexes navigate cellular barriers, such as the nuclear envelope or cytoskeletal structures, after assembly, a spatial dimension must be integrated into F3CS, an unmet technical challenge that currently limits our understanding of trimeric signaling.

Submitted October 27, 2025, and accepted for publication March 3, 2026.
*Correspondence: elizabeth.hinde@unimelb.edu.au
Editor: Sudipta Maiti.
https://doi.org/10.1016/j.bpj.2026.03.007
© 2026 The Authors. Published by Elsevier Inc. on behalf of Biophysical Society.
This is an open access article under the CC BY license (http://creativecommons.org/licenses/by/4.0/).

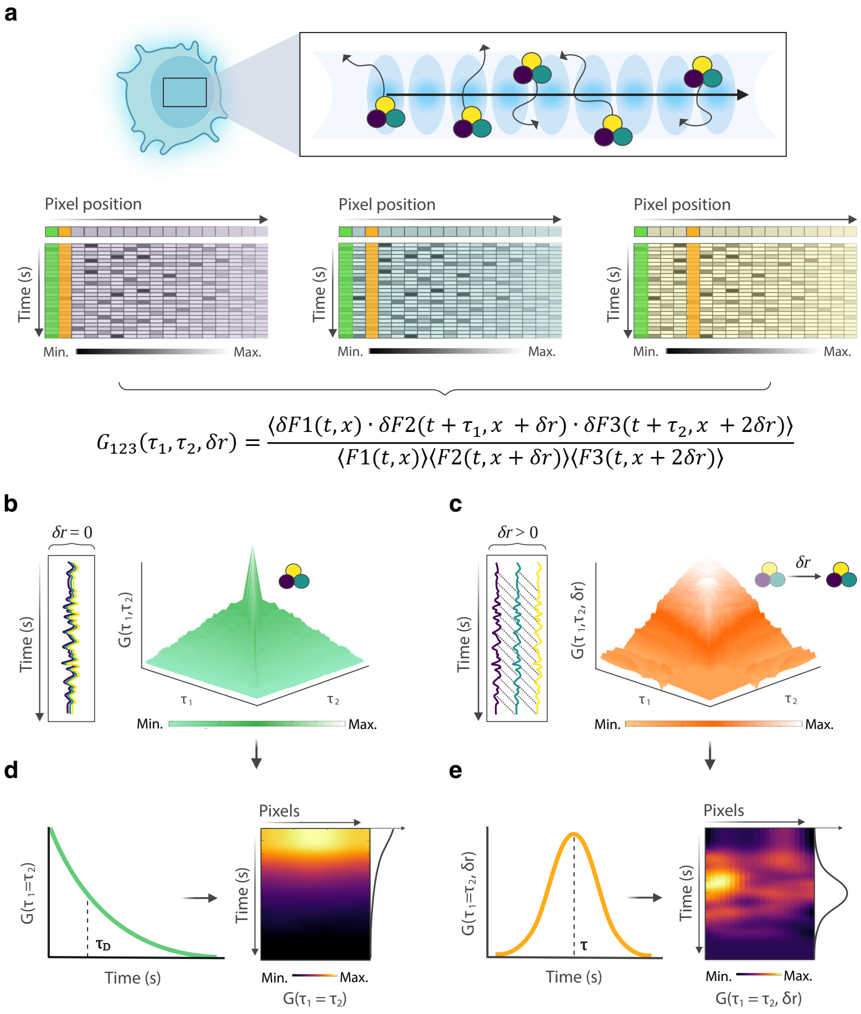

FIGURE 1 Schematic overview of spatial triple-correlation spectroscopy (S3CS). (*a–c*) S3CS resolves short- to long-range diffusion of fluorescent heterotrimers across a three-channel scan (*a*), by triple-cross-correlation of the spectrally distinct fluorescence fluctuations recorded: 1) within each pixel ($\delta r = 0$) (*b*) versus 2) between spatially offset pixels ($\delta r > 0$) (*c*); across two time delays (τ_1 and τ_2). In each case, this calculation gives rise to a 2D triple-correlation surface that reports heterotrimer concentration and dynamics across short- to long-range spatial scales. (*d* and *e*) By extracting the diagonal $G_{123}(\tau_1 = \tau_2)$ (*d*) or $G_{123}(\tau_1 = \tau_2, \delta r)$ (*e*) from each 2D triple-correlation surface and constructing them into carpets, S3CS enables direct readout of heterotrimer compartment-specific arrival times and thus a spatiotemporal map of heterotrimer transport throughout living cells.

FCS, in its original form, is a single-point live-cell microscopy method that quantifies the local diffusion coefficient of a fluorescently labeled protein as a function of its interaction with one or more spectrally distinct species.[19–22] This is achieved through a one-, two-, or three-channel acquisition of fluorescence intensity fluctuations as proteins pass through the fixed observation volume of a one- or two-photon laser scanning microscope, followed by temporal auto-, cross, or triple-correlation analysis within and between channels.[18,23,24] While multichannel single-point FCS can detect heterodimeric and heterotrimeric interactions at specific subcellular sites, it is spatially restricted, offering only a localized view of diffusion and failing to capture how intracellular compartmentalization or physical barriers influence molecular transport before or after the molecule enters the detection volume. To overcome this limitation, pair correlation microscopy was developed.[25,26] This FCS-based method replaces the static point measurement with a line- or frame-scanned acquisition[27] and incorporates spatial information directly into the analysis via use of a pair correlation function (pCF) that temporally compares fluorescence fluctuations between spatially offset pixels.[25,26] The result is a matrix of correlation values that reveals how molecular transport evolves relative to intracellular structures such as the actin cytoskeleton,[28] nuclear envelope,[29–32] or chromatin.[33–37] Although pCF has been successfully adapted for heterodimer analysis using two-channel scans and cross-pCF functions,[26,32,34] no current framework supports its extension to heterotrimers. While three-channel line-scanning is technically feasible, the lack of a spatial analog to the triple-correlation function used in F3CS has thus far prevented application in this context.

To address this technological gap and enable spatiotemporal tracking of heterotrimer dynamics in live cells, here we introduce a new method that integrates a spatial triple-correlation function into the framework of pair correlation microscopy (Fig. 1). This method, termed spatial triple-correlation spectroscopy (S3CS), combines a three-channel line-scan acquisition with a spatial triple-correlation

function that compares fluorescence fluctuations from three spectrally distinct molecular species across two temporal delays (τ_1 and τ_2) and a defined spatial offset (δr). The result is a series of triple-correlation surfaces that capture heterotrimer diffusion pathways relative to intracellular barriers and compartments. These surfaces can be collapsed into correlation carpets that spatially map changes in arrival time and transport efficiency, while also resolving heterotrimer assembly and disassembly events in live cells. We demonstrate the utility of S3CS in two biologically relevant systems: first, by applying it to importin-α/importin-β/cargo-mediated nucleocytoplasmic transport,[29] where it resolves the formation and disassembly of the complex across the nuclear envelope; and, second, by tracking the NF-Y transcription factor,[38] where it reveals that trimeric assembly precedes chromatin binding. Together, these findings establish S3CS as a powerful method for mapping heterotrimer dynamics in live cells, filling a key methodological gap and providing new insight into the spatial regulation of protein signaling.

METHODS

Simulations

To first test the ability of S3CS to selectively detect heterotrimer transport in silico, we simulated three-channel confocal line scans (64 pixels spanning 3.2 μm) using a three-dimensional (3D) Gaussian point spread function (PSF) (radial waist = 0.25 μm; axial waist = 1.5 μm) sampling a 6.4-μm square box populated with 200 particles per channel diffusing freely in the *x-y* plane (diffusion coefficient, D = 10 $\mu m^2\ s^{-1}$). The 64-pixel line was scanned 300,000 times at 1953 Hz, and each particle exhibited a molecular brightness of 100,000 counts s^{-1} molecule^{-1} that appeared in one (monomers), two (heterodimers), or three (heterotrimers) of the scan's channels. The resulting three-channel 64 × 300,000-pixel intensity carpets served as negative (monomers and heterodimers) and positive (heterotrimers) controls to assess the specificity of S3CS. To next explore the ability of S3CS to quantify heterotrimer concentration (particle number) and mobility (diffusion coefficient) during a transport event in silico, we again simulated three-channel 64 × 300,000 confocal line scans as described above, but this time the box was populated exclusively with heterotrimeric particles (i.e., 100,000 counts s^{-1} molecule^{-1} in all three channels). Two conditions were examined: 1) varying particle number (N = 30–400) at a fixed diffusion rate (D = 10 $\mu m^2\ s^{-1}$) and 2) a fixed particle number (N = 200) with varying diffusion rates (D = 1–20 $\mu m^2\ s^{-1}$). To further test S3CS performance under heterogeneous conditions, heterotrimers were then combined with monomers at defined abundance ratios of 50:50, 70:30, or 90:10 while maintaining a constant total particle number of 200. This design enabled assessment of whether the triple-correlation fraction (Q_{123}) could accurately recover the relative contribution of heterotrimers in mixed populations. Finally, to investigate S3CS sensitivity to spatially heterogeneous diffusion, particles were simulated with D = 25 $\mu m^2\ s^{-1}$ in the presence of a trapping zone that imparted D = 1.25 $\mu m^2\ s^{-1}$. The trapping zone (0.32 μm wide) was simulated as either permeable (entry/exit probabilities = 0.2) or impermeable (entry/exit probabilities = 0), thereby imparting anisotropy in transport across the scanned region. All simulations were generated using SimFCS software (Laboratory for Fluorescence Dynamics, University of California, Irvine; https://www.lfd.uci.edu/globals/).

Multiple-tau approach to S3CS calculation

To efficiently compute spatial triple-correlation functions across three-channel line scan data in the time domain and over the broad temporal range required to capture heterotrimer dynamics in living cells, we adapted the multiple-τ correlation algorithm traditionally used for one- or two-channel autocorrelation function (ACF) and cross-correlation function (CCF) analyses[39–45] for application to S3CS. Within this framework, the spatial triple-correlation function is defined as Eq. 1:

$$G_{123}\left(\tau_1, \tau_2, \delta r\right) = \frac{\langle \delta F1(t,x) \cdot \delta F2(t+\tau_1, x+\delta r) \cdot \delta F3(t+\tau_2, x+2\delta r)\rangle}{\langle F1(t,x)\rangle \langle F2(t,x+\delta r)\rangle \langle F3(t,x+2\delta r)\rangle}, \quad (1)$$

where $F1(t,x)$, $F2(t,x+\delta r)$, and $F3(t,x+2\delta r)$ denote the fluorescence intensities as functions of time t at pixel positions x, $x+\delta r$, and $x+2\delta r$, respectively, and δr is the spatial shift between pixels (i.e., an integer number of pixels). The corresponding fluctuations $\delta F1(t,x)$, $\delta F2(t+\tau_1,x+\delta r)$ and $\delta F3(t+\tau_2,x+2\delta r)$, represent the deviations of the fluorescence intensities as functions of time from their respective mean values (e.g., $\delta F1(t,x) = F1(t,x) - \langle F1(t,x)\rangle$), evaluated at lag times τ_1 and τ_2 for the shifted pixel positions $x+\delta r$ and $x+2\delta r$, respectively. The lag times (τ_1 and τ_2) are defined by the multiple-τ correlation algorithm binning scheme, in which short-lag correlations are computed at the native temporal resolution (i.e., integer multiples of the sampling time) to preserve fine temporal detail, while longer-lag correlations are evaluated using progressively coarser bins. Within each bin, intensity traces are averaged, and the triple-correlation function is recalculated iteratively with the effective bin width doubling at each step, to provide accurate correlation estimates across a wide temporal range while minimizing redundant computation. Collectively, this formulation produces, for each pixel ($\delta r = 0$, corresponding to the scanning equivalent of F3CS) or pixel triplet ($\delta r > 0$), a full 2D triple-correlation surface $G_{123}(\tau_1,\tau_2,\delta r)$ that forms the basis for subsequent S3CS analysis and triple-correlation carpet construction. For $\delta r > 0$, direction-dependent transport can also be assessed by reversing the spatial ordering of the pixel triplet (i.e., $G_{321}(\tau_1,\tau_2,\delta r)$), enabling direct comparison of forward and reverse dynamics.

S3CS analysis and triple-correlation carpet construction

For detection and spatial mapping of heterotrimer transport, the S3CS 2D correlation surfaces computed at each pixel or pixel triplet were filtered and organized to preserve the temporal ordering required for interpretation under a Markov assumption.[18] Specifically, we imposed the condition $\tau_1 \leq \tau_2$, ensuring that correlation events are temporally ordered and causally interpretable—a constraint that results in correlation values being considered over a triangular (τ_1, τ_2) domain. For visualization, correlation values for which $\tau_1 > \tau_2$ were mapped to their symmetric counterparts such that $G_{123}(\tau_1, \tau_2, \delta r) = G_{123}(\tau_2, \tau_1, \delta r)$, yielding a visually symmetric 2D correlation matrix when indexed by τ_1 and τ_2. To enable spatial mapping along line scans, we then extracted the diagonal $\tau_1 = \tau_2$ from each filtered 2D correlation matrix. This diagonal preserves the characteristic transport timing of correlated fluctuations while reducing the data to a 1D correlation profile per spatial position. The resulting profiles were assembled into triple-correlation carpets, in which each column corresponds to a position along the scanned line and the vertical axis represents lag time on a logarithmic scale. Although this study focuses on the $\tau_1 = \tau_2$ diagonal for intuitive spatial visualization and quantitative analysis of direction-dependent transport along line scans, the full 2D triple-correlation surfaces provided by the underlying multi-τ formulation prior to filtering preserve the potential of S3CS to investigate asynchronous or sequential interaction dynamics in future applications.

Extracting heterotrimer dynamics from S3CS carpets

To elucidate the diffusion dynamics of heterotrimeric protein complexes, $G_{123}(\tau_1 = \tau_2)$ profiles derived from simulation and live cell experiments were fit with a one-component model function for Brownian diffusion in a 3D Gaussian PSF according to Eq. 2:[17]

$$G_{123}(\tau_1 = \tau_2) = A^2\left(1+\frac{4\tau}{3\tau_D}\right)^{-1}\left(1+\frac{4\tau}{3\omega_0^2\tau_D}\right)^{-1/2}+y_0, \tag{2}$$

where A is the amplitude; τ_D is the diffusion time, which is defined as $\frac{\omega_0^2}{4D}$, where D is the diffusion coefficient; τ is the lag time; ω_0 is the confocal beam radial waist (which is calibrated by single point FCS measurement of fluorescent dye standards with known D and calculation of the spectral average[23]); and y_0 is a baseline correction term (offset). While for $G_{123}(\tau_1 = \tau_2, \delta r)$ profiles, those derived from simulation were fit to the same model function defined by Eq. 2 but with a diffusion propagator altered to consider δr,[27] and those derived from live cell experiments were fit to a Gaussian probability distribution with a characteristic peak arrival time (τ), since intracellular diffusion across a scale greater than the confocal PSF is highly anisotropic.[26] Nonlinear least squares was used to fit and extract correlation amplitudes (i.e., G_{123} (0,0), G_{123} (max($\tau_1 = \tau_2$, δr)) as well as characteristic decay and arrival times (τ_D and τ).

Extracting heterotrimer fraction from S3CS carpets

Triple-correlation amplitudes are related to the number of ternary complexes present.[16,17] It has been established from triple colour coincidence analysis that the number of ternary complexes (N_{123}) within the confocal volume can be derived from the triple-correlation amplitude ($G_{123}(0,0)$) and the corresponding single-channel autocorrelation amplitudes ($G_1(0)$,$G_2(0)$,$G_3(0)$) as $\langle N_{123}\rangle = G_{123}(0,0)/(G_1(0)G_2(0)G_3(0))$.[16] Therefore, to assess the proportion of ternary complexes present, a relative fraction (Q_{123}) was calculated by normalizing S3CS amplitudes to the single-channel ACF amplitudes, according to Eq. 3:

$$Q_{123} = \frac{\gamma_3}{\gamma_2^2}\times max\left(\frac{G_{123}(0,0)}{G_1(0)\times G_2(0)},\frac{G_{123}(0,0)}{G_1(0)\times G_3(0)},\frac{G_{123}(0,0)}{G_2(0)\times G_3(0)}\right), \tag{3}$$

where $G_{123}(0,0)$ denotes the maximum S3CS amplitude; $G_1(0)$, $G_2(0)$, and $G_3(0)$ represent the maximum amplitudes of the ACFs for the first, second, and third channels; and γ_3 and γ_2 are correction factors accounting for the effective observation volumes of triple and double correlations (see results).

Cell culture and plasmid construction

HeLa cells (ATCC, catalog no. CCL-2) were grown in Dulbecco's modified Eagle's medium-high glucose (DMEM) (D5796, Sigma-Aldrich, Missouri, USA) supplemented with 10% (vol/vol) fetal bovine growth serum (Gibco, Massachusetts, USA), 100 U mL^{-1} penicillin, and 100 μg mL^{-1} streptomycin (PS; 15,140-122, Gibco). Cells were maintained in a humidified 5% CO_2 atmosphere at 37°C and passaged every 2 or 3 days, not exceeding 15 passages. For confocal imaging, the cells were seeded on 35-mm glass-bottom culture dishes (0.17 ± 0.1 mm bottom glass thickness) (FD35-100, WPI, Florida, USA) and grown to 70–80% confluence over 2 days. Routine mycoplasma testing was performed to ensure mycoplasma-free conditions throughout the study. The plasmids encoding eGFP, mCherry, Halo, and NLS-eGFP are available on Addgene. The pmCherry-NF-YA, peGFP-NF-YAm29, pmCherry-NF-YAm29, peGFP-NF-YB, and pmCherry-NF-YB plasmids were previously described.[46] For all subsequent cloning, standard PCR with custom-designed primers was performed, followed by digestion using fast-digest restriction enzymes and ligation with T4 DNA ligase as per the manufacturer's instructions. Briefly, to obtain the eGFP-Halo-mCherry control construct, mCherry and Halo were amplified from their respective plasmids, digested with *Xho*I + *Hin*dIII and *Hin*dIII + *Pst*I, and subsequently cloned into a peGFP-C1 vector previously digested with *Xho*I + *Pst*I. For the establishment of the peGFP-C1-Halo, the peGFP-C1 vector was digested with *Hin*dIII and *Pst*I and ligated to a similarly digested Halo PCR product. The pmCherry-C1-Halo construct was made by adding the *Kpn*I- and *Bam*HI-digested Halo PCR fragment into the pmCherry-C1 vector digested with *Kpn*I and *Bam*HI. To clone pHalo-KPNA2, KPNA2 was amplified from the pEN_TT 3xFLAP-KPNA2 plasmid (192306, Addgene) and inserted into pHalo after double digestion with *Kpn*I and *Bam*HI. Likewise, to clone pmCherry-KPNB1, KPNB1 was amplified from the peGFP-N1_importin_beta plasmid (106941, Addgene) and inserted into pmCherry-C1 after digestion with *Sac*I and *Bam*HI. All vectors were transformed into chemically competent *Escherichia coli* DH5α cells (Thermo Fisher, Massachusetts, USA) and selected on Lysogeny broth agar plates. All constructs were confirmed by Sanger sequencing (AGRF, Melbourne, Australia) before use. All enzymes and reagents were sourced from New England Biolabs (Victoria, Australia).

Plasmid transfection for live-cell imaging and halo-tagged protein staining

To transfect plasmids for live-cell imaging, Lipofectamine 3000 Transfection Reagent (L3000015, Invitrogen, California, USA) was used as per the manufacturer's instructions. The typical transfection mixture for a 35-mm glass-bottom dish with 80% cell confluence consisted of 1 μg plasmid, 2.5 μL P3000, and 3 μL Lipofectamine 3000. Details on the specific plasmids used for each transfection type are provided in Table 1.

The Halo tag dye Janelia Fluor (JF) 646 (G1002, Promega, Wisconsin, USA) was reconstituted in dimethyl sulfoxide from lyophilized powder to prepare a 100-μM stock solution. HeLa cells transiently expressing Halo fusion proteins were subsequently labeled with 100 nM JF 646 diluted in DMEM medium at 37°C for 15 min. Unbound JF646 was removed prior to imaging by confocal laser scanning microscopy by washing the cells with DMEM buffer supplemented with 10% (vol/vol) fetal bovine growth serum, 100 U mL^{-1} penicillin, and 100 μg mL^{-1} streptomycin.

TABLE 1 Plasmid transfection for S3CS analysis using live-cell data

Assay	Plasmid concentration	Related figures
Negative control 1	0.3 μg eGFP + 0.3 μg mCherry + 0.3 μg Halo	Figs. 4 and 6
Negative control 2	0.3 μg eGFP-Halo + 0.3 μg mCherry-Halo + 0.3 μg mCherry-eGFP	Figs. 4 and 6
Positive control	1 μg eGFP-mCherry-Halo	Fig. 4
NLS/importin-α/β	0.3 μg NLS-eGFP + 0.3 μg KPNA2-Halo + 0.3 μg KPNB1-Halo	Fig. 5
NF-Y	0.3 μg eGFP-NF-YB or eGFP-NF-YAm29 + 0.3 μg mCherry-NF-YA or mCherry-NF-YB + 0.3 μg NF-YC-Halo	Fig. 6

Confocal laser scanning microscopy

All live-cell S3CS three-channel line scan experiments were performed on a Zeiss 880 confocal laser scanning microscope equipped with a Plan-Apochromat 63× (1.2 NA) water-immersion objective and an environmental chamber for temperature and CO_2 control (37°C, 5% CO_2). For three-channel excitation and detection of eGFP, mCherry, and Halo-JF646 construct emissions, the 488-nm argon, 561-nm diode, and 633-nm He-Ne laser lines were employed simultaneously and at low power (~1 μW at the objective) to minimize photobleaching (<10% across line scan acquisition). The resulting fluorescent signals were directed through a 488/561/633 dichroic mirror to one GaAsP and two PMT detectors set to collect 500–550 nm (eGFP), 600–650 nm (mCherry), and 670–756 nm (Halo-JF646) to minimize spectral bleed-through (<5% between pairs of channels). For three-channel line scan recording of eGFP, mCherry, and Halo-JF646 construct dynamics, HeLa cells exhibiting low expression of each fluorescent protein (<100 nM) were selected and then rapid line scans were acquired across subcellular regions of interest using high electronic zoom (3.4-μm), low pixel frame size (16 × 1 pixels, 210-nm pixel size) and short pixel dwell time (7.81 μs) to minimize the line time (0.293 ms) and maximize statistics (>300,000 lines). Collectively, these parameters suppressed spectral crosstalk, accounted for the inverse-square scaling of triple-correlation amplitudes with the number of fluorescent complexes in the observation volume ($G_{123} \propto 1/N^2$), and ensured sufficient detection of coincident events for S3CS analysis with adequate signal/noise ratio (SNR) and statistical convergence. The resulting three-channel line scans were exported as TIFF files and analyzed in MATLAB.

Live-cell line scan data preprocessing

All live-cell three-channel line scan data upon import into MATLAB were first pre-processed via use of a temporally segmented ACF analysis to eliminate artifacts related to long-term instability (e.g., photobleaching) and transient but bright macromolecular events (e.g., vesicle movement).[12,47–51] Briefly, the time series from each channel was divided into temporal segments (20,000 lines), and the ACF of each segment was determined separately via use of a fast Fourier transformation algorithm based on the Wiener-Khinchin theorem to enhance computational efficiency. Segments displaying ACFs significantly deviating from the average, particularly those with substantial photobleaching or cell movement artifacts (typically less than 3% of all segments), were identified and excluded via a general statistical test based on their standard deviation. Specifically, any segment whose ACF exhibited a standard deviation exceeding three times the mean standard deviation, a user-defined threshold, was systematically discarded. This approach effectively filtered out anomalies, preserving data integrity. Crucially, to maintain consistent temporal alignment across all channels, any segment removed from one channel was also excluded from the other channels. The remaining segments, then become the input data for subsequent S3CS analysis, triple-correlation carpet construction, and extraction of heterotrimer dynamics.

RESULTS

In silico testing of the principle behind S3CS

To establish the conceptual basis for S3CS, we initially employed simulations to demonstrate that a triple-cross-correlation function can: 1) selectively detect the transport of heterotrimers between three spatially distinct observation volumes and 2) yield an analytical output capable of tracking this coordinated movement across a microscope-scanned acquisition, particularly when visualized as a triple-correlation carpet. This involved two key steps. First, we simulated three-channel line scan microscopy datasets based on a 64-pixel line with 3.2 μm total length (50 nm per pixel), scanned up to 500,000 times at a sampling frequency of 1953 cycles s^{-1}, using a 3D Gaussian PSF-defined observation volume with a 250 nm radial waist, which captured fluorescence intensity fluctuations arising from the diffusion of homogeneous and heterogeneous particle populations of: 1) monomers (independently present in each channel), 2) heterodimers (simultaneously present in two channels), and 3) heterotrimers (present in all three channels); under a range of biophysical conditions, including diffusion coefficients ($D = 1$–20 μm^2 s^{-1}), particle numbers ($N = 30$–400 per channel), and interspecies abundance ratios (30:70, 50:50, 90:10), with or without diffusion barriers (see methods). Then, second, we explored a computational strategy for triple-cross-correlating spectrally distinct and spatially offset signals within the intensity carpets generated by each simulation.

To do so, we first focused on simulated datasets representing a homogeneous population of either monomers, heterodimers, or heterotrimers ($N = 200$ particles per channel) undergoing isotropic diffusion ($D = 10$ μm^2 s^{-1}), and tested the computational efficiency as well as specificity of a multiple-τ algorithm at calculating triple-cross-correlation functions capable of tracking heterotrimer short- to long-range diffusion across three-channel line scans. For each dataset, this involved cross-correlating three spectrally distinct signals across two temporal delays (τ_1, τ_2), first within individual pixels (analogous to F3CS; Fig. 2, *a*–c), and then between pixels separated by a spatial offset (δr, in pixels) greater than the simulated PSF radial waist (i.e., $\delta r > 5$ or > 250 nm) (Fig. 2, *d*–*f*). In the latter case (i.e., S3CS), the triple correlation is evaluated across three pixels at positions x (the reference pixel), $x + \delta r$, and $x + 2\delta r$, where δr defines the step between adjacent pixels and the total spatial span of the correlation is $2\delta r$. Collectively, these calculations yield, for each pixel or pixel triplet, a 2D triple-correlation surface, which is then averaged across the scan to obtain a representative 2D correlation surface for each condition (Fig. 2, *b* and *e*). Comparison of the resulting 2D correlation surfaces' 1D diagonals (i.e., $\tau_1 = \tau_2$) at $\delta r = 0$ (Fig. 2 *c*) and $\delta r = 6$ (300 nm) (Fig. 2 *f*) revealed positive-amplitude triple-correlation profiles exclusively for the heterotrimer condition, demonstrating that S3CS extends the capacity of F3CS to specifically detect heterotrimer diffusion across spatial scales beyond a single observation volume. This result is supported by: 1) control analyses showing selective detection of monomers and heterodimers by single-channel autocorrelation and two-channel cross-correlation, respectively (Fig. S1), and 2) the systematic evolution of the heterotrimer $\tau_1 = \tau_2$ diagonals with increasing spatial offsets from $\delta r = 0$ to 6 (Fig. S2).

Next, we tested whether the $\tau_1 = \tau_2$ diagonals of the 2D S3CS correlation surfaces could serve as 1D readouts for

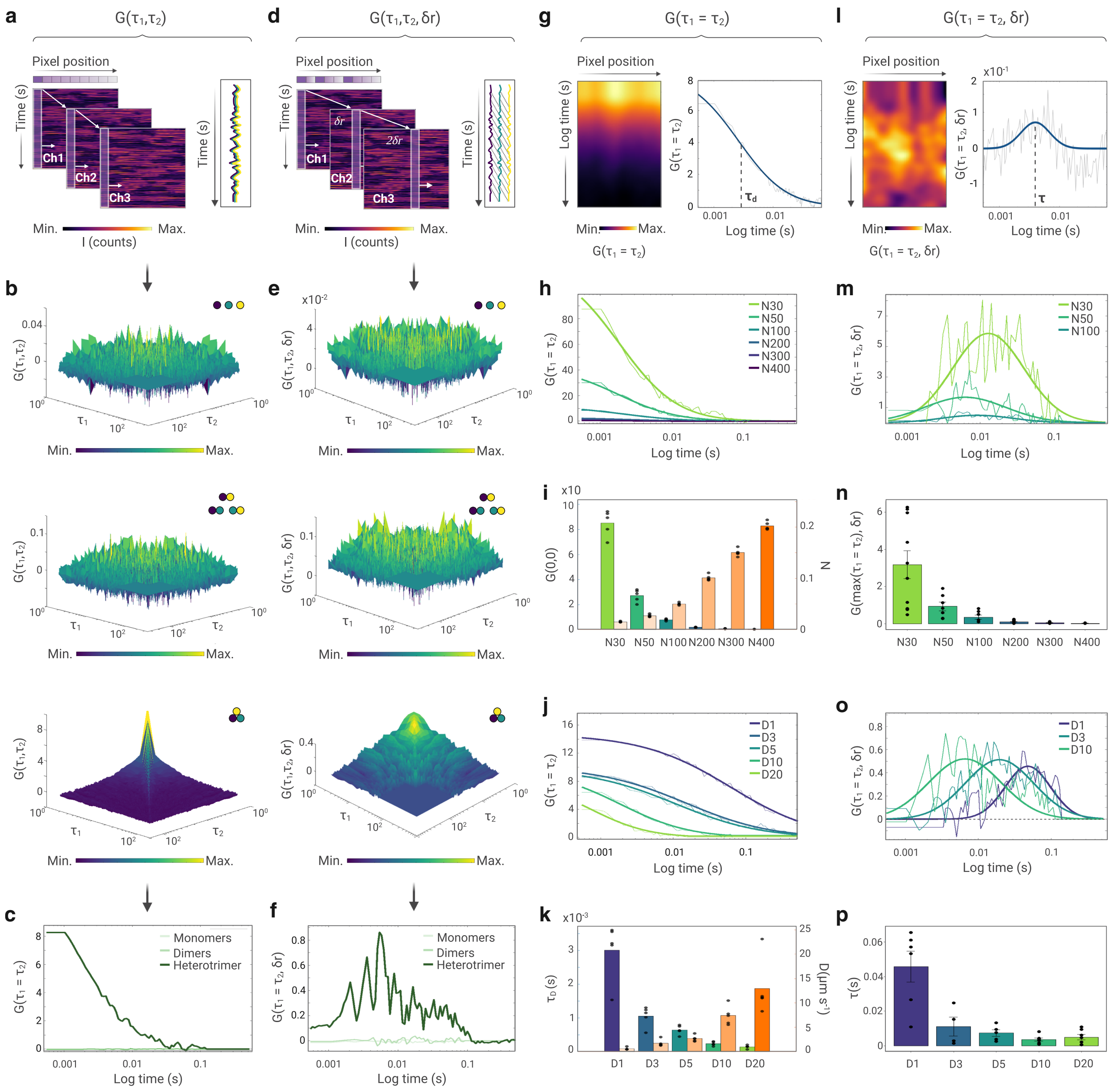

FIGURE 2 S3CS extracts simulated heterotrimeric complex concentration and dynamics. (*a*) Schematic of S3CS analysis triple correlating the spectrally distinct fluorescence fluctuations recorded in each pixel of a simulated three-channel line scan at $\delta r = 0$. (*b*) Representative average 2D triple-correlation surfaces ($\delta r = 0$) that result from simulations considering a homogeneous population of monomers (*top*), dimers (*middle*), and heterotrimers (*bottom*) undergoing isotropic diffusion ($N = 200$, $D = 10\,\mu m^2\,s^{-1}$). (*c*) $\tau_1 = \tau_2$ diagonals, $G_{123}(\tau_1 = \tau_2)$, extracted from the 2D triple-correlation surfaces in (*b*). (*d*) Schematic of S3CS analysis at $\delta r > 0$. (*e*) Representative average 2D triple-correlation surfaces ($\delta r = 6$) from the simulations in (*b*). (*f*) $G_{123}(\tau_1 = \tau_2, \delta r = 6)$ profiles extracted from the 2D triple-correlation surfaces in (*e*). (*g*) Representative $G_{123}(\tau_1 = \tau_2)$ profile from an S3CS carpet ($\delta r = 0$), with an amplitude $G_{123}(0,0)$, which scales with the number of complexes (N) and a decay that reflects the molecular diffusion time (τ_d). (*h* and *i*) Global fits of $G_{123}(\tau_1 = \tau_2)$ profiles (*h*) and the corresponding number of complexes within the observation volume (in *orange*) (*i*) from simulations of heterotrimers diffusing isotropically ($N = 30$–400, $D = 10\,\mu m^2\,s^{-1}$). (*j* and *k*) Global fits of $G_{123}(\tau_1 = \tau_2)$ profiles (*j*) and corresponding diffusion coefficient (in *orange*) (*k*) from simulations with fixed $N = 100$ and varying D (1–20 $\mu m^2\,s^{-1}$). (*l*) Representative $G_{123}(\tau_1 = \tau_2, \delta r)$ profile from an S3CS carpet ($\delta r = 6$), with a maximum correlation amplitude ($G_{123}(\max(\tau_1 = \tau_2, \delta r))$ at the characteristic arrival time (τ). (*m* and *n*) Global fits of $G_{123}(\tau_1 = \tau_2, \delta r = 6)$ profiles (*m*) and their maximum amplitudes (*n*) from the datasets in (*h*). (*o* and *p*) Global fits of $G_{123}(\tau_1 = \tau_2, \delta r = 6)$ profiles (*o*) and their characteristic arrival times (*p*) from the datasets in (*j*). Fitted curves in (*h*) and (*j*) were obtained using a one-component diffusion model for a 3D Gaussian PSF, while fits in (*m*) and (*o*) used a general Gaussian model. Data in (*i*), (*k*), (*n*), and (*p*) are shown as mean ± SE.

visualizing heterotrimer diffusion across pixel and pixel triplet positions in a microscope scan, from short ($\delta r = 0$) (Fig. 2, *g*–*k*) to long ($\delta r = 6$) (Fig. 2, *l*–*p*) spatial ranges. To this end, we analyzed simulated datasets representing homogeneous populations of heterotrimers undergoing isotropic diffusion, either at a fixed diffusion rate with varying particle numbers

($D = 10\ \mu m^2\ s^{-1}$; N = 30–400), or at varying diffusion rates with a fixed particle number (N = 200; $D = 1–20\ \mu m^2\ s^{-1}$), and assessed whether the temporal profiles of the resulting 1D S3CS diagonals quantitatively reflected the simulated parameters when assembled into triple-correlation carpets. This analysis involved fitting the S3CS diagonals calculated at $\delta r = 0$ ($G_{123}(\tau_1 = \tau_2)$, Fig. 2 *g*) and at $\delta r > 0$ ($G_{123}(\tau_1 = \tau_2, \delta r)$, Fig. 2 *l*) to a one-component diffusion model, using a propagator modified to account for δr, since the simulated dynamics were isotropic (see methods).

From these fits, we extracted: 1) the maximum amplitude of each correlation profile (i.e., $G_{123}(0,0)$ or $G_{123}(\max(\tau_1 = \tau_2, \delta r))$ (Fig. 2, *h* and *m*), which in the case of $G_{123}(0,0)$ scales with the apparent number of complexes (N) according to $G_{123(0,0)} \propto \frac{\gamma_3}{N^2}$, where γ_3 is a correction factor that accounts for the effective triple-correlation observation volume (Fig. S3 *a*) and 2) the characteristic decay time (τ_D), which for the $G_{123}(\tau_1 = \tau_2)$ profile enables quantification of the local diffusion coefficient (D) (Fig. 2, *j* and *o*). Comparison of the fitted parameters (Figs. 2, *i–k*, *n*, *p* and S4) confirmed that both $G_{123}(\tau_1 = \tau_2)$ and $G_{123}(\tau_1 = \tau_2, \delta r)$ provide quantitative readouts of heterotrimer dynamics and, when arranged into triple-correlation carpets, enable spatiotemporal visualization of heterotrimer concentration and diffusion. It is important to note that, because the zero-lag triple-correlation amplitude of $G_{123}(\tau_1 = \tau_2)$ profiles scale inversely with the square of the apparent number of fluorescent complexes, reliable estimation of heterotrimer dynamics is restricted to low-expression regimes (<100 nM) as higher concentrations rapidly reduce the SNR (Fig. S5). In addition, robust statistical convergence of $G_{123}(\tau_1 = \tau_2, \delta r)$ profiles at nonzero spatial offsets require long acquisitions (>300,000 line scans; Fig. S6), reflecting the reduced probability of detecting coordinated fluorescence fluctuations across spatially separated observation volumes.

Next, we examined whether triple-correlation carpets derived from $G_{123}(\tau_1 = \tau_2)$ and $G_{123}(\tau_1 = \tau_2, \delta r)$ profiles remain quantitative readouts of heterotrimer dynamics under more complex, biologically relevant conditions, such as in heterogeneous molecular populations or spatially heterogeneous environments. To this end, we first analyzed simulated datasets in which heterotrimers underwent isotropic diffusion ($D = 10\ \mu m^2\ s^{-1}$) in the presence of increasing fractions of monomers (50:50, 30:70, and 10:90 ratios), and assessed whether, upon fitting to a one-component diffusion model, the relative fraction of heterotrimers (Q_{123}) could be extracted from the maximum correlation amplitude of the $G_{123}(\tau_1 = \tau_2)$ profiles ($G_{123}(0,0)$) and remain detectable within the $G_{123}(\tau_1 = \tau_2, \delta r)$ profiles. This analysis involved normalizing $G_{123}(0,0)$ against the two individual channel ACF amplitudes ($G_{11}(0)$, $G_{22}(0)$, $G_{33}(0)$) that maximized Q_{123} and thus accounted for the third individual channel containing the limiting number of molecules (Fig. 3, *a* and *b*). A comparison of the fit results after correction with γ_3 versus double-correlation volumes (γ_2^2) (Fig. S3 *a*) confirmed that Q_{123} values accurately reflected the simulated heterotrimer fractions (Fig. 3, *c* and *d*) and that this population remained detectable in recovered $G_{123}(\tau_1 = \tau_2, \delta r)$ profiles (Fig. 3, *e* and *f*).

Finally, we extended our analysis to simulated datasets in which heterotrimers diffused with $D = 25\ \mu m^2\ s^{-1}$ in the presence of permeable (0.2 probability) or nonpermeable (0 probability) barriers introducing anisotropy ($D = 1.25\ \mu m^2\ s^{-1}$) (Fig. 3 *h*), to test whether: 1) spatial heterogeneity in local heterotrimer diffusion (D) could be extracted from $G_{123}(\tau_1 = \tau_2)$ profile τ_D values fitted with a one-component diffusion model and 2) barrier permeability toward long-range heterotrimer diffusion could be recovered from $G_{123}(\tau_1 = \tau_2, \delta r)$ profile τ values fitted with a probability distribution function, since isotropic transport could no longer be assumed at $\delta r > 0$ (see methods). To do so, we first inspected triple-correlation carpets constructed from $G_{123}(\tau_1 = \tau_2)$, identified barrier locations from the lengthened correlation profiles reflecting reduced diffusion (Fig. 3 *i*), and compared D values inside versus outside these zones by fitting average $G_{123}(\tau_1 = \tau_2)$ profiles (Fig. 3, *j* and *k*). We then applied the same procedure to carpets constructed from $G_{123}(\tau_1 = \tau_2, \delta r)$, identifying regions influenced by barrier permeability (Fig. 3 *l*) and analyzing τ values to assess long-range transport (Fig. 3, *m* and *n*). Collectively, these analyses showed that, while $G_{123}(\tau_1 = \tau_2)$ carpets can report the presence of simulated barriers, only $G_{123}(\tau_1 = \tau_2, \delta r)$ carpets reveal differences in barrier permeability, an important regulator of heterotrimer diffusion. Thus, S3CS uniquely enables quantitative assessment of heterotrimer transport and access across spatially heterogeneous environments.

Live-cell validation of S3CS capacity to track heterotrimer transport

To validate that S3CS can specifically track heterotrimer transport with respect to subcellular architecture in living cells; and define a protocol for the acquisition of fluorescence fluctuation data amenable to this method of analysis; we next performed three-channel confocal line scan microscopy experiments across the cytoplasm and nucleus of live HeLa cells transiently transfected with: 1) eGFP-mCherry-Halo646 (a triple-colored heterotrimer), 2) eGFP-mCherry, mCherry-Halo646, eGFP-Halo646 (dual-colored heterodimers), or 3) eGFP, mCherry, Halo646 (single-colored monomers). This involved first selecting HeLa cells expressing these constructs at nanomolar levels (Fig. S7) and acquiring three-channel line scans across their nuclear envelopes (Fig. 4, *a* and *b*) under conditions that minimized spectral bleed-through (Figs. S8–S10) while providing sufficient spatiotemporal resolution to capture fluorescence

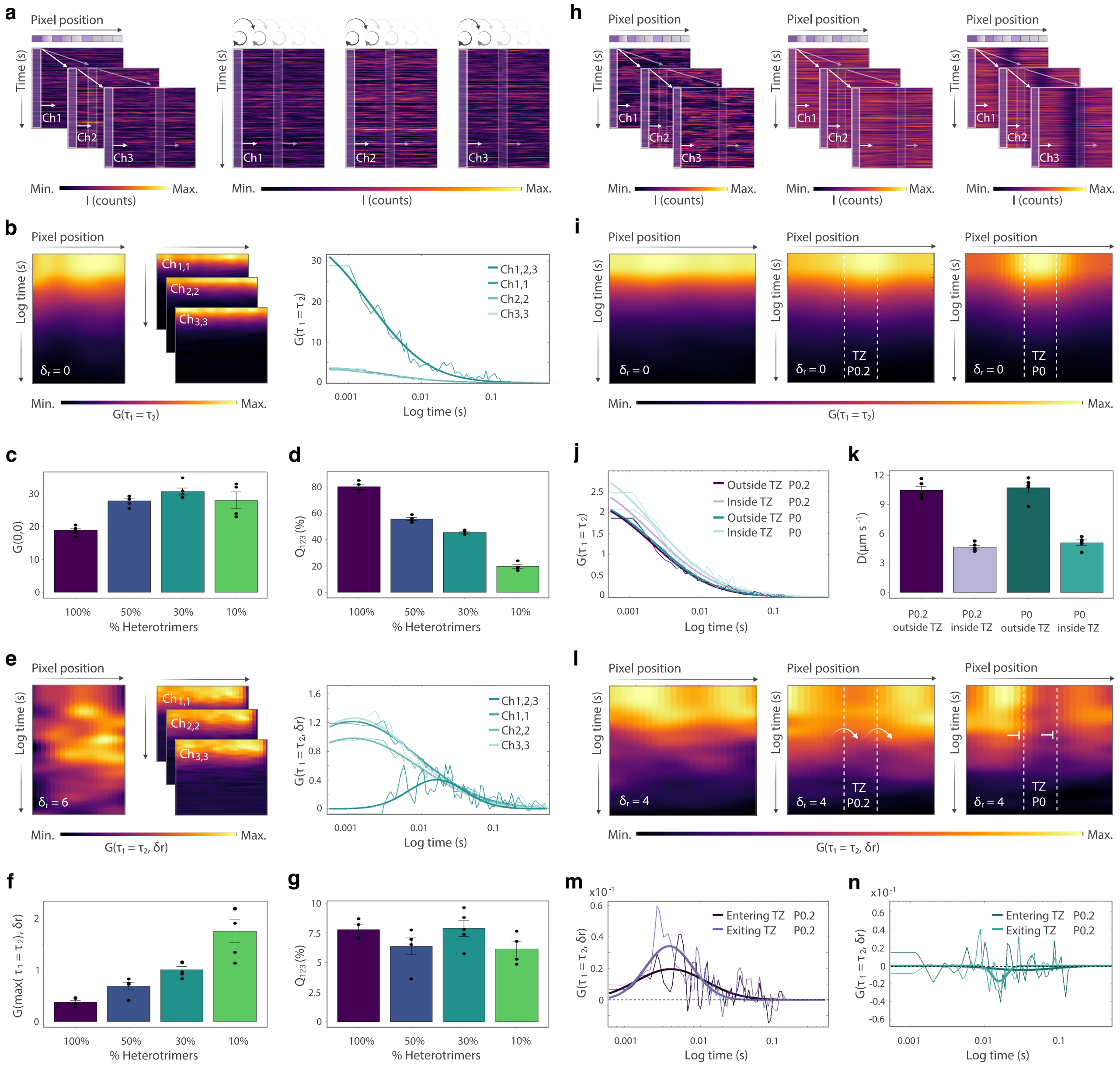

FIGURE 3 S3CS accurately quantifies triple-correlation fractions and delineates the anisotropic motions of simulated heterotrimeric complexes experiencing anomalous diffusion. (*a*) Schematic of S3CS and pair correlation function (pCF) analyses correlating spectrally distinct fluorescence fluctuations recorded in each pixel of a simulated three-channel line scan at $\delta r = 0$ and $\delta r > 0$. (*b*) Global fits of the average $\tau_1 = \tau_2$ diagonal ($G_{123}(\tau_1 = \tau_2)$) and pCF profiles (Ch1, Ch2, and Ch3) from S3CS and pCF carpets ($\delta r = 0$) for a simulation considering a heterogeneous population of heterotrimeric complexes and monomers ($N = 200$ in total, $D = 10\ \mu m^2\ s^{-1}$), with the heterotrimer fraction set to 30%. Bold lines indicate fitted curves. (*c*) Maximum $G_{123}(\tau_1 = \tau_2)$ amplitudes (i.e., $G_{123}(0,0)$) for simulations considering a heterogeneous population of heterotrimeric complexes and monomers ($N = 200$ and $D = 10\ \mu m^2\ s^{-1}$ for both populations), where heterotrimer fraction ranged from 100 to 10%. (*d*) Relative fraction of heterotrimers (Q_{123}) from the simulations in (*c*). Data are shown as mean $\pm$ SE. (*e*) Global fits of the average $\tau_1 = \tau_2$ diagonal ($G_{123}(\tau_1 = \tau_2, \delta r)$) and pCF profiles (Ch1, Ch2, and Ch3) from S3CS and pCF carpets ($\delta r = 6$) for the simulation in (*b*). Bold lines indicate fitted curves. (*f* and *g*) Maximum $G_{123}(\tau_1 = \tau_2, \delta r = 6)$ amplitudes (*f*) and Q_{123} ($\delta r = 6$) (*g*) for the simulations in (*c*). Data are shown as mean $\pm$ SE. (*h*) Schematic of S3CS analysis triple correlating spectrally distinct fluorescence fluctuations recorded in each pixel of a simulated three-channel line scan at $\delta r = 0$ and $\delta r > 0$, with complexes diffusing isotropically (*left*) or in the presence of a trapping zone (TZ) (*center* and *right*). (*i*) S3CS carpets ($\delta r = 0$) from simulations considering a homogeneous population of complexes ($N = 200$) in the absence or presence of a central TZ. Diffusion coefficients inside and outside the TZ were 1.25 and 25 $\mu m^2\ s^{-1}$, with entry/exit probabilities of 0.2 (*center*) or 0 (*right*). (*j*) Global fits of $G_{123}(\tau_1 = \tau_2)$ profiles inside and outside the TZ. (*k*) Diffusion coefficients from fitted $G_{123}(\tau_1 = \tau_2)$ profiles from the simulations in (*i*). (*l*) S3CS carpets ($\delta r = 4$) from the simulations in (*i*). (*m* and *n*) Global fits of $G_{123}(\tau_1 = \tau_2, \delta r = 4)$ profiles for complexes entering and exiting the TZ. Fitted curves in (*b*) and (*j*) were obtained using a one-component diffusion model for a 3D Gaussian PSF, while fits in (*e*), (*m*), and (*n*) used a general Gaussian model.

fluctuations from single-protein complexes (see methods). The simulation-derived S3CS analytical workflow (Figs. 2 and 3) was then applied to this experimentally acquired line scan data (Fig. 4, *c–f*) in a temporally segmented manner (see methods), which collectively enabled slow-timescale artifacts inherent to live-cell data acquisition to be filtered out while preserving the fast-timescale dynamics of heterotrimer transport across a spatially heterogeneous environment.

A comparison of the averaged 2D S3CS correlation surfaces calculated at $\delta r = 0$ from three-channel line scan data recording fluorescent heterotrimer versus heterodimer and monomer dynamics in live cells (Fig. 4 *c*), alongside an overlay of the corresponding 1D $G_{123}(\tau_1 = \tau_2)$ profiles (Fig. 4 *d*), revealed that only heterotrimers produce a positive $G_{123}(0,0)$ amplitude. Quantification of this observation, through calculation of the relative fraction of heterotrimer Q_{123} present

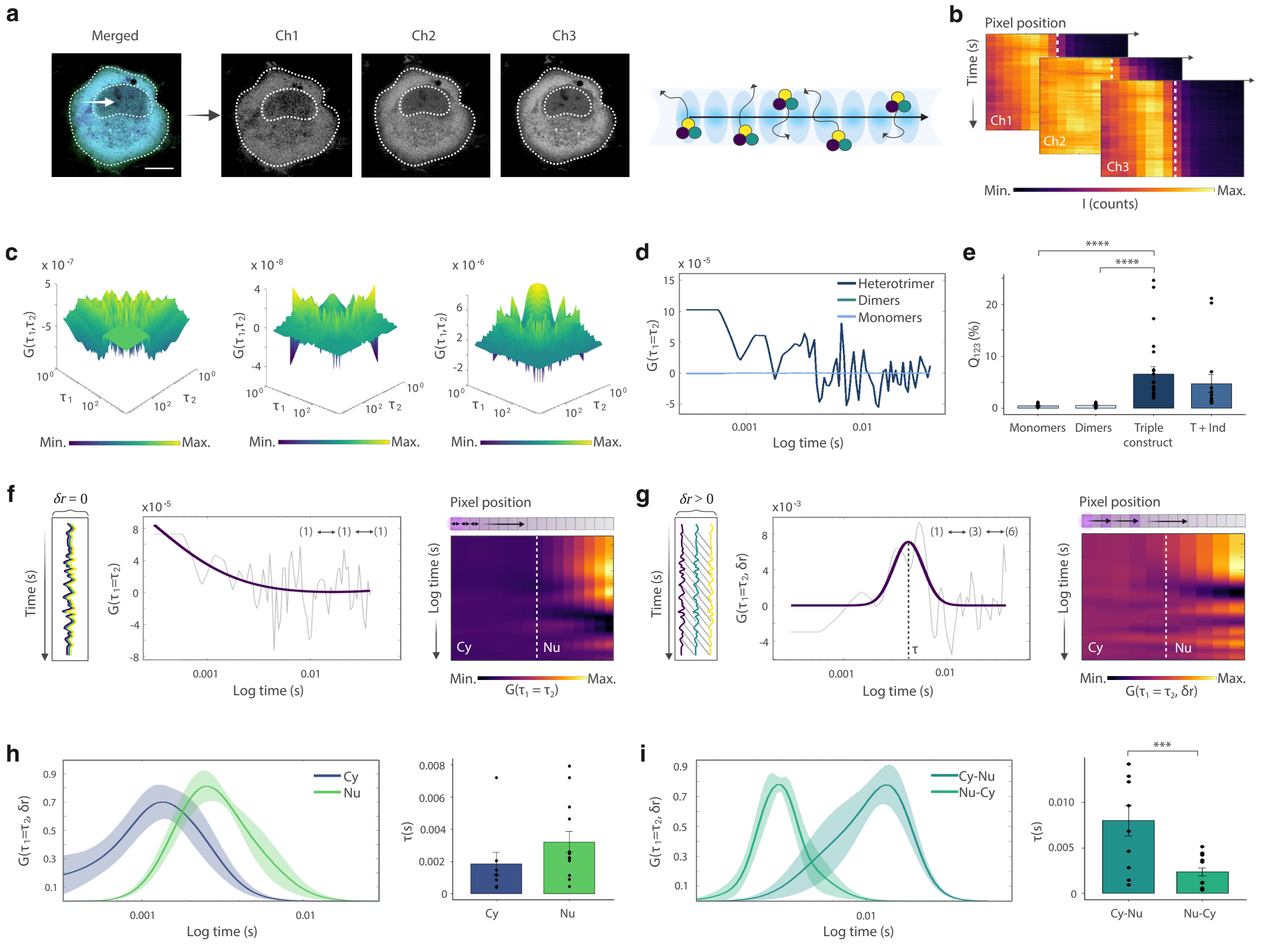

FIGURE 4 S3CS analysis reveals the diffusive behavior of heterotrimeric protein complexes within subcellular environments. (*a*) Representative confocal image of a HeLa cell transfected with the eGFP-mCherry-Halo646 triple construct. A three-color merged image is shown on the left; single-channel images for eGFP (Ch1), mCherry (Ch2), and Halo646 (Ch3) are shown on the right. The line across the nuclear envelope indicates the direction of the three-channel line scan. Scale bar, 11 μm. (*b*) Fluorescence intensity fluctuations arising from eGFP-mCherry-Halo646 diffusion in and out of pixels along the line scan are plotted as intensity carpets, with the *x* axis denoting pixel position and the *y* axis denoting time. (*c*) Representative average 2D triple-correlation surfaces from HeLa cells cotransfected with monomers (eGFP, mCherry, and Halo646) (*left*), dimers (eGFP-mCherry, eGFP-Halo646, and mCherry-Halo646) (*center*), or the eGFP-mCherry-Halo646 triple construct (*right*). (*d*) $G_{123}(\tau_1 = \tau_2)$ diagonals extracted from the correlation surfaces in (*c*). (*e*) Relative fraction of heterotrimers (Q_{123}) from the datasets in (*c*) and cells cotransfected with the triple construct and monomers ($n = 10$ [monomers], $n = 10$ [dimers], $n = 23$ [triple construct], $n = 15$ [triple construct + monomers]). (*f*) Representative average $G_{123}(\tau_1 = \tau_2)$ profile from an S3CS carpet ($\delta r = 0$), with an amplitude that, relative to the autocorrelation amplitudes in the two limiting channels, is indicative of Q_{123}. (*g*) Representative average $G_{123}(\tau_1 = \tau_2, \delta r)$ profile from an S3CS carpet ($\delta r = 3$), with a peak time reflecting the complex's characteristic arrival time (τ). (*h*) Global fits of $G_{123}(\tau_1 = \tau_2, \delta r = 3)$ profiles for eGFP-mCherry-Halo646 mobility in the cytoplasm (Cy) and nucleus (Nu), with corresponding transit times on the right ($n = 9$ [Cy], $n = 13$ [Nu]). (*i*) Global fits of $G_{123}(\tau_1 = \tau_2, \delta r = 3)$ and $G_{321}(\tau_1 = \tau_2, \delta r = 3)$ profiles for eGFP-mCherry-Halo646 mobility across the nuclear envelope, with transit times on the right ($n = 10$ [Cy to Nu], $n = 16$ [Nu to Cy]). All $G_{123}(\tau_1 = \tau_2, \delta r)$ profiles were normalized between 0 and 1. Shading indicates mean $\pm$ SE. Data in (*e*), (*h*), and (*i*) are shown as mean $\pm$ SE. Significant differences were determined by unpaired Student's *t*-test (***$p \leq 0.001$ and ****$p \leq 0.0001$). The fitted curve in (*f*) was obtained using a one-component diffusion model for a 3D Gaussian PSF, while fits in (*g*)–(*i*) used a general Gaussian model.

across multiple experiments (Fig. 4 *e*), demonstrates that S3CS is specific for the detection of heterotrimer dynamics in live cells (Q_{123} = 4.78 ± 1.71). Construction of the 2D S3CS correlation surface diagonals calculated at δr ≥ 0 from three-channel line scan data recording fluorescent heterotrimer dynamics into $G_{123}(\tau_1 = \tau_2)$ versus $G_{123}(\tau_1 = \tau_2, \delta r = 3$ or 630 nm) triple-correlation carpets (Fig. 4, *f* and *g*) revealed a spatial heterogeneity in this construct's subcellular concentration (see $G_{123}(0,0)$ values in Fig. 4 *f*) that appeared to result from delayed transport across the nuclear envelope (see τ values in Fig. 4 *g*). Quantification of this observation, through a fit-based extraction of the heterotrimer's bidirectional transit times τ within (τ_{CY} = 0.18 ± 0.07 ms and τ_{NU} = 0.32 ± 0.06 ms) and between ($\tau_{CY\text{-}NU}$ = 7.9 ± 0.17 ms and $\tau_{NU\text{-}CY}$ = 2.4 ± 0.4 ms) the cell cytoplasm and nucleus (Figs. 4, *h*, *i* and S11), demonstrates that S3CS can spatiotemporally track heterotrimer transport with respect to intracellular barriers such as the nuclear envelope.

To further establish the capacity of S3CS to resolve direction-dependent heterotrimer transport, we applied the acquisition and bidirectional analysis workflow, previously optimized for inert heterotrimer dynamics (Fig. 4, *f–j*), to the canonical NLS cargo/importin-α/importin-β nuclear import system. This involved transient transfection of HeLa cells with NLS-eGFP (cargo), Halo-KPNA2 (importin-α), and mCherry-KPNB1 (importin-β) (Fig. 5 *a*), and acquisition of three-channel line scan data across the nuclear envelope of cells exhibiting nanomolar expression levels of these three constructs, for direction-dependent S3CS analysis (Fig. 5 *b*). In the cytoplasm, it is reported that importin-α binds NLS cargo and recruit's importin-β to form a heterotrimer that traverses the nuclear pore, where Ran-GTP binding in the nucleus dissociates the complex and releases the cargo (Fig. 5 *c*). Consistent with this mechanism, positive-amplitude 2D S3CS surfaces were observed only in the cytoplasm (Fig. 5 *d*), and construction of $G_{123}(\tau_1 = \tau_2, \delta r = 3$ or 630 nm) triple-correlation carpets revealed

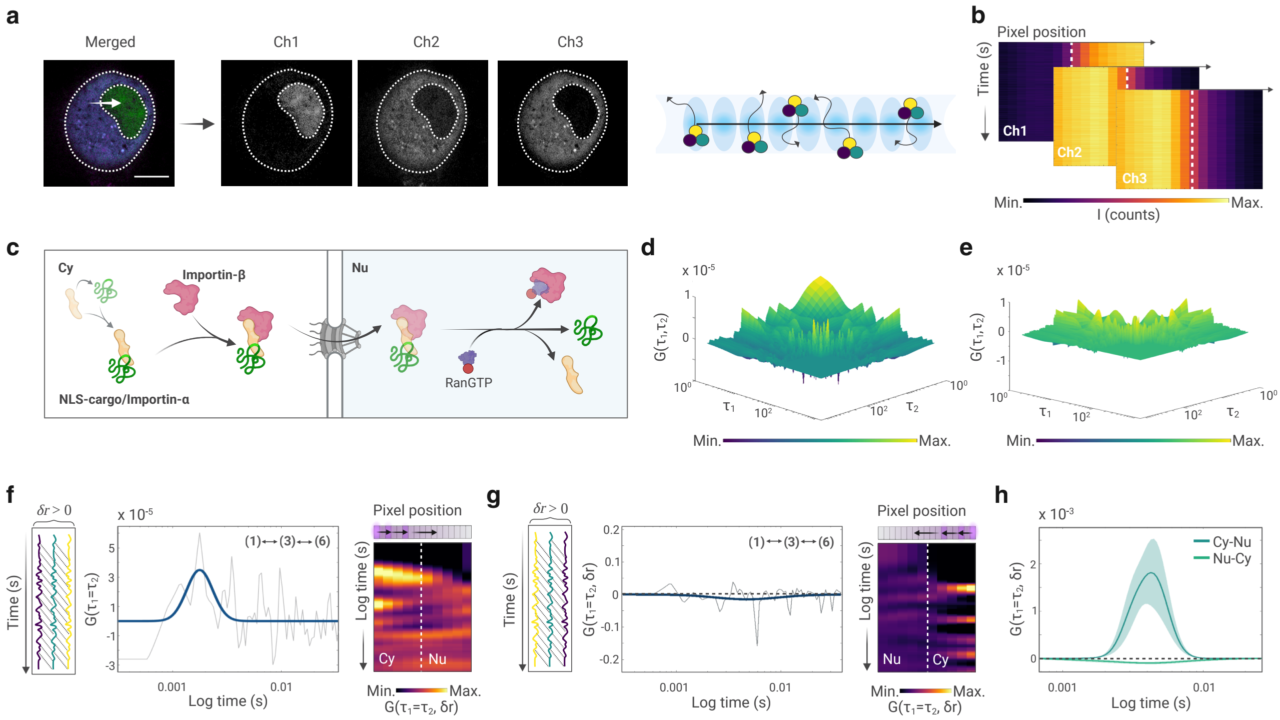

FIGURE. 5 S3CS elucidates reversible interactions between heterotrimeric protein subunits. (*a*) Representative confocal image of a HeLa cell cotransfected with eGFP coupled to a nuclear localization signal (NLS), mCherry-KPNB1 (importin-β), and Halo646-KPNA2 (importin-α). A three-color merged image is shown on the left; single-channel images for NLS-eGFP (Ch1), mCherry-KPNB1 (Ch2), and Halo646-KPNA2 (Ch3) are shown on the right. The line across the nuclear envelope indicates the orientation of the three-channel line scan. Scale bar, 11 μm. (*b*) Fluorescence intensity fluctuations from eGFP-NLS-mCherry-KPNB1-Halo646-KPNA2 diffusion in and out of pixels along the line scan are plotted as intensity carpets, with the *x* axis denoting pixel position and the *y* axis denoting time. (*c*) Schematic overview of the NLS-cargo-importin-α/β system. In the classical nuclear import pathway, importin-α binds proteins bearing an NLS and recruits importin-β, enabling translocation of the heterotrimeric complex across the nuclear pore complex. Dissociation inside the nucleus is triggered by Ran-GTP binding to importin-β. (*d* and *e*) Representative 2D triple-correlation surfaces in the cytoplasm (Cy) (*d*) and nucleus (Nu) (*e*) from the cell shown (*a*). (*f* and *g*) Representative average $G_{123}(\tau_1 = \tau_2, \delta r)$ profile from an S3CS carpet (δr = 3), with a peak time reflecting the complex's characteristic arrival time (τ). In (*g*), the analysis was performed by reversing the line scan's orientation (i.e., G_{321} ($\tau_1 = \tau_2$, δr = 3)). (*h*) Global fits of $G_{123}(\tau_1 = \tau_2, \delta r = 3)$ and $G_{321}(\tau_1 = \tau_2, \delta r = 3)$ profiles reporting heterotrimer mobility from Cy to Nu (*blue*) and from Nu to Cy (*green*). Shading indicates mean ± SE. Data shown as mean ± SE.

heterotrimer transport exclusively in the cytoplasm-to-nucleus direction (Fig. 5, *f–h*). Quantification of this observation via transit time analysis τ ($\tau_{CY\text{-}NU} = 2.1 \pm 0.6$ ms) (Figs. 5 *h* and S11) demonstrates S3CS's ability to discriminate irreversible from reversible transport processes across compartments.

Having validated S3CS in both simulated (Figs. 2 and 3) and controlled live-cell systems that demonstrate its ability to resolve direction-dependent transport with respect to intracellular architecture (Figs. 4 and 5), we next applied it to the NF-Y transcription factor complex that, according to biochemical and structural studies, relies on heterotrimer formation between its YA, YB, and YC subunits,[52–54] for high fidelity control of transcription initiation in protein-coding genes featuring the CCAAT box within their promoters.[55–57] This involved transfecting HeLa cells with mCherry-NF-YA, eGFP-NF-YB, and Halo-NF-YC (Fig. 6 *a*), and acquisition of three-channel line scan data across the nucleoplasm of cells expressing these constructs at nanomolar levels (Fig. 6 *b*) for S3CS analysis (Fig. 6, *c* and *d*). Positive-amplitude 2D S3CS surfaces were observed in the nucleoplasm (Fig. 6 *e*), and quantification of the fraction of NF-Y heterotrimer from their corresponding 1D $G_{123}(\tau_1 = \tau_2)$ profiles via calculation of Q_{123} across multiple experiments (Fig. 6 *f*), enabled the first live-cell detection of the NF-Y heterotrimer ($Q_{123} = 3.78 \pm 1.28$). To next probe the role of DNA binding in NF-Y heterotrimer formation, we repeated S3CS analysis with a dominant-negative NF-YA mutant (NF-Yam29), which cannot engage the CCAAT box but still associates with NF-YB and NF-YC.[56] Consistent with prior reports, from $G_{123}(\tau_1 = \tau_2)$ and $G_{123}(\tau_1 = \tau_2, \delta r = 3 \text{ or } 630 \text{ nm})$ analysis we found that NF-Yam29 was still incorporated into a stable heterotrimeric NFY complex at levels comparable with wild type ($Q_{123} = 5.12 \pm 1.64$) (Fig. 6 *g*), while exhibiting greater mobility as a result of loss in DNA engagement ($\tau_{NF\text{-}Y} = 4.1 \pm 0.9$ ms and $\tau_{NF\text{-}Yam29} = 2.2 \pm 0.2$ ms)

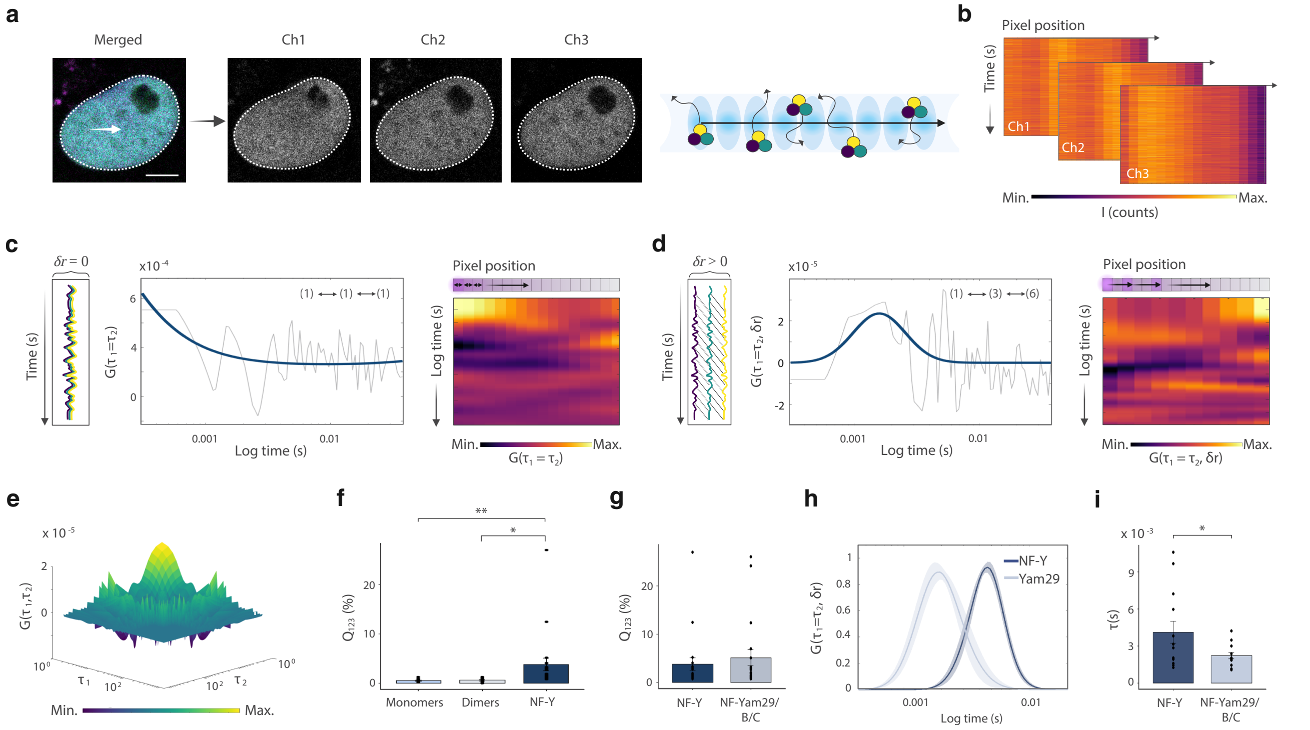

FIGURE 6 S3CS directly demonstrates the formation of ternary NF-Y complexes. (*a*) Representative confocal image of a HeLa cell cotransfected with eGFP-NF-YB, mCherry-NF-YA, and NF-YC-Halo646. A three-color merged image is shown on the left; single-channel images for eGFP-NF-YB (Ch1), mCherry-NF-YA (Ch2), and NF-YC-Halo646 (Ch3) are shown on the right. The line across the nucleus indicates the orientation of the three-channel line scan. Scale bar, 6 μm. (*b*) Fluorescence intensity fluctuations from eGFP-NF-YB-mCherry-NF-YA-NF-YC-Halo646 diffusion in and out of pixels along the line scan are plotted as intensity carpets, with the *x* axis denoting pixel position and the *y* axis denoting time. (*c*) Representative $G_{123}(\tau_1 = \tau_2)$ profile from an S3CS carpet ($\delta r = 0$) with an amplitude that, relative to the autocorrelation amplitudes in the two limiting channels, is indicative of the relative fraction of heterotrimers (Q_{123}). (*d*) Representative $G_{123}(\tau_1 = \tau_2, \delta r)$ profile from an S3CS carpet ($\delta r = 3$), with a peak time indicative of the complex's characteristic arrival time (τ). (*e*) Representative 2D triple-correlation surfaces for the cell shown in (*a*). (*f*) Q_{123} from cells cotransfected with monomers (eGFP, mCherry, and Halo646), dimers (eGFP-mCherry, eGFP-Halo646, and mCherry-Halo646), or the NF-Y subunits (eGFP-NF-YB, mCherry-NF-YA, and NF-YC-Halo646) ($n = 10$ [monomers], $n = 10$ [dimers], $n = 21$ [NF-Y]). (*g*) Q_{123} from cells cotransfected with the NF-Y subunits (*blue*) ($n = 21$) or eGFP-NF-YAm29, mCherry-YB, and Halo646-NF-YC (*light blue*) ($n = 20$). (*h*) Global fit of $G_{123}(\tau_1 = \tau_2, \delta r = 3)$ profiles reporting NF-YA/B/C and NF-Yam29/B/C mobilities in the nucleus. $G_{123}(\tau_1 = \tau_2, \delta r = 3)$ profiles were normalized between 0 and 1. Shading indicates mean $\pm$ SE. (*i*) Transit times from h ($n = 12$ [NF-YA/B/C], $n = 17$ [NF-Yam29/B/C]). Data in (*f*), (*g*), and (*i*) are shown as mean $\pm$ SE. Significant differences were determined by unpaired Student's *t*-test (*$p \leq 0.05$ and **$p \leq 0.01$). The fitted curve in (*c*) was obtained using a one-component diffusion model for a 3D Gaussian PSF, while fits in (*d* and *h*) used a general Gaussian model.

(Figs. 6, *h*, *i* and S11). Collectively, these results establish S3CS as a powerful tool for quantifying heterotrimer assembly, probing subunit-specific functions in live cells.

DISCUSSION

Here, we introduce S3CS, a novel approach for spatiotemporally resolving heterotrimer dynamics in live cells. By integrating a spatial dimension into triple-correlation analysis, S3CS extends the capabilities of conventional F3CS and pair correlation microscopy,[17,26] enabling direct visualization of heterotrimer transport relative to intracellular architecture. Our simulations demonstrate that S3CS selectively detects heterotrimers across a range of diffusion coefficients, particle numbers, and abundance ratios, while remaining quantitative under conditions of molecular heterogeneity and in the presence of spatial barriers. Importantly, the method differentiates local diffusion from long-range transport, highlighting its capacity to report on both intracellular confinement and direction-dependent permeability across structural obstacles such as the nuclear pore complex. Although we focus here on the $\tau_1 = \tau_2$ diagonal to enable spatial mapping along line scans, the underlying multi-τ, two-lag formulation preserves the full 2D triple-correlation surface, ensuring that S3CS remains applicable to asynchronous or sequential interaction dynamics in future studies. Live-cell experiments validate these predictions, showing that S3CS specifically identifies heterotrimeric assemblies while excluding monomeric or heterodimeric species. Application to the importin-α/importin-β/NLS cargo complex illustrates that S3CS can resolve direction-dependent transport across the nuclear envelope,[31] capturing transit times and confirming irreversible nuclear import in real time. This demonstrates the method's potential for probing transport kinetics and directionality in complex intracellular environments.

We further applied S3CS to the biologically relevant NF-Y transcription factor,[46] revealing heterotrimer assembly and subunit-specific contributions. The reduction in transit time for the NF-YA DNA-binding mutant indicates that S3CS can detect subtle alterations in kinetics linked to functional states or regulatory cues. These findings emphasize the method's versatility in dissecting both stable and transient protein assemblies, as well as interactions between distinct heterotrimeric complexes and other cellular factors. Collectively, our results establish S3CS as a powerful tool for mapping heterotrimeric protein dynamics in live cells. Beyond providing quantitative measures of diffusion, concentration, and transport, S3CS enables mechanistic insights into how protein complexes navigate cellular compartments, respond to environmental cues, and coordinate molecular interactions. The ability to track heterotrimer formation, disassembly, and interaction with other proteins opens new avenues for understanding the spatial regulation of signaling, transcription, and genome maintenance. Future applications of S3CS could extend to other multi-protein complexes, integrating with super-resolution and SPAD array detection[58] or functional imaging to link heterotrimer dynamics to cellular phenotype, ultimately enhancing our understanding of the molecular choreography underpinning cellular physiology.

DATA AND CODE AVAILABILITY

The custom MATLAB code for S3CS described in this study is publicly available on GitHub at: https://github.com/ehinde/Spatial-triple-correlation.

ACKNOWLEDGMENTS

We thank Prof. Thorsten Wohland for the thoughtful discussion of the results and methods throughout the duration of this work. We thank Prof. Roberto Mantovani and Andrea Bernardini for providing fluorescent constructs and advice on the generation of new constructs. E.H. was supported by an ARC Future Fellowship (FT200100401) and Jacob Haimson Beverly Mecklenburg Lectureship. This work was supported by ARC Discovery Project Grants (DP180101387 and DP21010298) and the ARC Centre of Excellence in Quantum Biotechnology (CE230100021). We thank the Biological Optical Microscopy Platform, University of Melbourne, for enabling access to the Zeiss LSM880 confocal laser scanning microscope.

AUTHOR CONTRIBUTIONS

E.H. conceived the study. J.S.-V. and E.H. wrote the manuscript. J.S.-V. conducted the experiments. J.S.-V. and T.S. analyzed the data. X.Z. created and provided unique reagents.

DECLARATION OF INTERESTS

The authors declare no competing interests.

SUPPORTING MATERIAL

Supporting material can be found online at https://doi.org/10.1016/j.bpj.2026.03.007.

REFERENCES

1. Bancaud, A., S. Huet, ..., J. Ellenberg. 2009. Molecular crowding affects diffusion and binding of nuclear proteins in heterochromatin and reveals the fractal organization of chromatin. *EMBO J.* 28:3785–3798. https://doi.org/10.1038/emboj.2009.340.
2. Baum, M., F. Erdel, ..., K. Rippe. 2014. Retrieving the intracellular topology from multi-scale protein mobility mapping in living cells. *Nat. Commun.* 5:4494. https://doi.org/10.1038/ncomms5494.
3. Solano, A., J. Lou, ..., E. Hinde. 2022. Radial pair correlation of molecular brightness fluctuations maps protein diffusion as a function of oligomeric state within live-cell nuclear architecture. *Biophys. J.* 121:2152–2167. https://doi.org/10.1016/j.bpj.2022.04.030.
4. Hinde, E., E. Pandžić, ..., K. Gaus. 2016. Quantifying the dynamics of the oligomeric transcription factor STAT3 by pair correlation of molecular brightness. *Nat. Commun.* 7:11047. https://doi.org/10.1038/ncomms11047.
5. Eggeling, C., C. Ringemann, ..., S. W. Hell. 2009. Direct observation of the nanoscale dynamics of membrane lipids in a living cell. *Nature.* 457:1159–1162. https://doi.org/10.1038/nature07596.
6. Hennen, J., I. Angert, ..., J. D. Mueller. 2018. Investigating LINC Complex Protein Homo-oligomerization in the Nuclear Envelopes of Living Cells Using Fluorescence Fluctuation Spectroscopy.

Methods Mol. Biol. 1840:121–135. https://doi.org/10.1007/978-1-4939-8691-0_11.

7. Streit, M., M. Budiarta, …, G. Beliu. 2025. Fluorescent labeling strategies for molecular bioimaging. *Biophys. Rep.* 5:100200. https://doi.org/10.1016/j.bpr.2025.100200.
8. Liu, Z., L. D. Lavis, and E. Betzig. 2015. Imaging Live-Cell Dynamics and Structure at the Single-Molecule Level. *Mol. Cell.* 58:644–659. https://doi.org/10.1016/j.molcel.2015.02.033.
9. Benke, A., N. Olivier, …, S. Manley. 2012. Multicolor single molecule tracking of stochastically active synthetic dyes. *Nano Lett.* 12:2619–2624. https://doi.org/10.1021/nl301018r.
10. Cutler, P. J., M. D. Malik, …, K. A. Lidke. 2013. Multi-color quantum dot tracking using a high-speed hyperspectral line-scanning microscope. *PLoS One.* 8:e64320. https://doi.org/10.1371/journal.pone.0064320.
11. Huang, T., C. Phelps, …, X. Nan. 2018. Simultaneous Multicolor Single-Molecule Tracking with Single-Laser Excitation via Spectral Imaging. *Biophys. J.* 114:301–310. https://doi.org/10.1016/j.bpj.2017.11.013.
12. Dunsing, V., A. Petrich, and S. Chiantia. 2021. Multicolor fluorescence fluctuation spectroscopy in living cells via spectral detection. *eLife.* 10:e69687. https://doi.org/10.7554/eLife.69687.
13. Foust, D. J., and D. W. Piston. 2025. Measuring G protein activation by spectrally resolved imaging fluorescence fluctuation spectroscopy. *Biophys. J.* 124:3354–3365. https://doi.org/10.1016/j.bpj.2024.08.008.
14. Krieger, J. W., A. P. Singh, …, T. Wohland. 2015. Imaging fluorescence (cross-) correlation spectroscopy in live cells and organisms. *Nat. Protoc.* 10:1948–1974. https://doi.org/10.1038/nprot.2015.100.
15. Schrimpf, W., V. Lemmens, …, J. Hendrix. 2018. Crosstalk-free multi-color RICS using spectral weighting. *Methods.* 140–141:97–111. https://doi.org/10.1016/j.ymeth.2018.01.022.
16. Heinze, K. G., M. Jahnz, and P. Schwille. 2004. Triple-color coincidence analysis: One step further in following higher order molecular complex formation. *Biophys. J.* 86:506–516. https://doi.org/10.1016/S0006-3495(04)74129-6.
17. Ridgeway, W. K., D. P. Millar, and J. R. Williamson. 2012. Quantitation of ten 30S ribosomal assembly intermediates using fluorescence triple correlation spectroscopy. *Proc. Natl. Acad. Sci. USA.* 109:13614–13619. https://doi.org/10.1073/pnas.1204620109.
18. Ridgeway, W. K., D. P. Millar, and J. R. Williamson. 2012. The Spectroscopic Basis of Fluorescence Triple Correlation Spectroscopy. *J. Phys. Chem. B.* 116:1908–1919. https://doi.org/10.1021/jp208605z.
19. Elson, E. L., and D. Magde. 1974. Fluorescence correlation spectroscopy. I. Conceptual basis and theory. *Biopolymers.* 13:1–27. https://doi.org/10.1002/bip.1974.360130102.
20. Magde, D., E. Elson, and W. W. Webb. 1972. Thermodynamic Fluctuations in a Reacting System—Measurement by Fluorescence Correlation Spectroscopy. *Phys. Rev. Lett.* 29:705–708. https://doi.org/10.1103/PhysRevLett.29.705.
21. Magde, D., E. L. Elson, and W. W. Webb. 1974. Fluorescence correlation spectroscopy. II. An experimental realization. *Biopolymers.* 13:29–61. https://doi.org/10.1002/bip.1974.360130103.
22. Wohland, T., S. Maiti, and R. Macháň. 2020. An Introduction to Fluorescence Correlation Spectroscopy. IOP Publishing.
23. Bacia, K., S. A. Kim, and P. Schwille. 2006. Fluorescence cross-correlation spectroscopy in living cells. *Nat. Methods.* 3:83–89. https://doi.org/10.1038/nmeth822.
24. Berland, K. M., P. T. So, and E. Gratton. 1995. Two-photon fluorescence correlation spectroscopy: method and application to the intracellular environment. *Biophys. J.* 68:694–701. https://doi.org/10.1016/S0006-3495(95)80230-4.
25. Digman, M. A., and E. Gratton. 2009. Imaging barriers to diffusion by pair correlation functions. *Biophys. J.* 97:665–673. https://doi.org/10.1016/j.bpj.2009.04.048.
26. Sanchez-Velasquez, J., A. Solano, …, E. Hinde. 2025. Pair correlation microscopy of intracellular molecular transport. *Nat. Protoc.* 20:1651–1677. https://doi.org/10.1038/s41596-024-01097-6.
27. Ries, J., and P. Schwille. 2006. Studying slow membrane dynamics with continuous wave scanning fluorescence correlation spectroscopy. *Biophys. J.* 91:1915–1924. https://doi.org/10.1529/biophysj.106.082297.
28. Chiu, C. L., M. A. Digman, and E. Gratton. 2013. Measuring Actin Flow in 3D Cell Protrusions. *Biophys. J.* 105:1746–1755. https://doi.org/10.1016/j.bpj.2013.07.057.
29. Cardarelli, F. 2017. Time-resolved biophysical approaches to nucleocytoplasmic transport. *Comput. Struct. Biotechnol. J.* 15:299–306. https://doi.org/10.1016/j.csbj.2017.03.005.
30. Cardarelli, F., and E. Gratton. 2010. In Vivo Imaging of Single-Molecule Translocation through Nuclear Pore Complexes by Pair Correlation Functions. *Biophys. J.* 98:751a. https://doi.org/10.1016/j.bpj.2009.12.4114.
31. Cardarelli, F., L. Lanzano, and E. Gratton. 2012. Capturing directed molecular motion in the nuclear pore complex of live cells. *Proc. Natl. Acad. Sci. USA.* 109:9863–9868. https://doi.org/10.1073/pnas.1200486109.
32. Hinde, E., K. Thammasiraphop, …, K. Gaus. 2017. Pair correlation microscopy reveals the role of nanoparticle shape in intracellular transport and site of drug release. *Nat. Nanotechnol.* 12:81–89. https://doi.org/10.1038/nnano.2016.160.
33. Hinde, E., X. Kong, …, E. Gratton. 2014. Chromatin dynamics during DNA repair revealed by pair correlation analysis of molecular flow in the nucleus. *Biophys. J.* 107:55–65. https://doi.org/10.1016/j.bpj.2014.05.027.
34. Lou, J., D. G. Priest, …, E. Hinde. 2020. Spatiotemporal dynamics of 53BP1 dimer recruitment to a DNA double strand break. *Nat. Commun.* 11:5776. https://doi.org/10.1038/s41467-020-19504-3.
35. Hinde, E., F. Cardarelli, …, E. Gratton. 2010. In vivo pair correlation analysis of EGFP intranuclear diffusion reveals DNA-dependent molecular flow. *Proc. Natl. Acad. Sci. USA.* 107:16560–16565. https://doi.org/10.1073/pnas.1006731107.
36. Hinde, E., F. Cardarelli, …, E. Gratton. 2012. Changes in chromatin compaction during the cell cycle revealed by micrometer-scale measurement of molecular flow in the nucleus. *Biophys. J.* 102:691–697. https://doi.org/10.1016/j.bpj.2011.11.4026.
37. Hinde, E., F. Cardarelli, …, E. Gratton. 2011. The Impact of Mitotic versus Interphase Chromatin Architecture on the Molecular Flow of EGFP by Pair Correlation Analysis. *Biophys. J.* 100:1829–1836. https://doi.org/10.1016/j.bpj.2011.02.024.
38. Manni, I., G. Mazzaro, …, G. Piaggio. 2001. NF-Y mediates the transcriptional inhibition of the promoters upon induced G arrest. *J. Biol. Chem.* 276:5570–5576. https://doi.org/10.1074/jbc.M006052200.
39. Laurence, T. A., S. Fore, and T. Huser. 2006. Fast, flexible algorithm for calculating photon correlations. *Opt. Lett.* 31:829–831. https://doi.org/10.1364/Ol.31.000829.
40. Magatti, D., and F. Ferri. 2001. Fast multi-tau real-time software correlator for dynamic light scattering. *Appl. Opt.* 40:4011–4021. https://doi.org/10.1364/Ao.40.004011.
41. Magatti, D., and F. Ferri. 2003. 25 ns software correlator for photon and fluorescence correlation spectroscopy. *Rev. Sci. Instrum.* 74:1135–1144. https://doi.org/10.1063/1.1525876.
42. Müller, P. 2012. Python Multiple-Tau Algorithm (Version 0.4.1). https://pypi.python.org/pypi/multipletau/.
43. Wohland, T., R. Rigler, and H. Vogel. 2001. The standard deviation in fluorescence correlation spectroscopy. *Biophys. J.* 80:2987–2999. https://doi.org/10.1016/S0006-3495(01)76264-9.
44. Bevington, P., and D. K. Robinson. 2003. Data Reduction and Error Analysis for Physical Sciences, 3rd edn. McGraw-Hill Education, pp. 67–71.
45. Di Rienzo, C., F. Cardarelli, …, E. Gratton. 2016. Diffusion Tensor Analysis by Two-Dimensional Pair Correlation of Fluorescence

Fluctuations in Cells. *Biophys. J.* 111:841–851. https://doi.org/10.1016/j.bpj.2016.07.005.

46. Priest, D. G., A. Bernardini, …, E. Hinde. 2021. Live cell dynamics of the NF-Y transcription factor. *Sci. Rep.* 11:10992. https://doi.org/10.1038/s41598-021-90081-1.

47. Dunsing, V., and S. Chiantia. 2018. A fluorescence fluctuation spectroscopy assay of protein-protein interactions at cell-cell contacts. *JoVE.* 142:e58582. https://doi.org/10.3791/58582.

48. Ridgeway, W. K., D. P. Millar, and J. R. Williamson. 2013. Vectorized data acquisition and fast triple-correlation integrals for Fluorescence Triple. *Comput. Phys. Commun.* 184:1322–1332. https://doi.org/10.1016/j.cpc.2012.12.022.

49. Kohler, J., K. H. Hur, and J. D. Mueller. 2023. Autocorrelation function of finite-length data in fluorescence correlation spectroscopy. *Biophys. J.* 122:241–253. https://doi.org/10.1016/j.bpj.2022.10.027.

50. Waithe, D., F. Schneider, …, C. Eggeling. 2018. Optimized processing and analysis of conventional confocal microscopy generated scanning FCS data. *Methods.* 140–141:62–73. https://doi.org/10.1016/j.ymeth.2017.09.010.

51. Kolin, D. L., S. Costantino, and P. W. Wiseman. 2006. Sampling effects, noise, and photobleaching in temporal image correlation spectroscopy. *Biophys. J.* 90:628–639. https://doi.org/10.1529/biophysj.105.072322.

52. Fleming, J. D., G. Pavesi, …, K. Struhl. 2013. NF-Y coassociates with FOS at promoters, enhancers, repetitive elements, and inactive chromatin regions, and is stereo-positioned with growth-controlling transcription factors. *Genome Res.* 23:1195–1209. https://doi.org/10.1101/gr.148080.112.

53. Nardini, M., N. Gnesutta, …, R. Mantovani. 2013. Sequence-Specific Transcription Factor NF-Y Displays Histone-like DNA Binding and H2B-like Ubiquitination. *Cell.* 152:132–143. https://doi.org/10.1016/j.cell.2012.11.047.

54. Nardone, V., A. Chaves-Sanjuan, and M. Nardini. 2017. Structural determinants for NF-Y/DNA interaction at the CCAAT box. *Biochim. Biophys. Acta. Gene Regul. Mech.* 1860:571–580. https://doi.org/10.1016/j.bbagrm.2016.09.006.

55. Huber, E. M., D. H. Scharf, …, A. A. Brakhage. 2012. DNA Minor Groove Sensing and Widening by the CCAAT-Binding Complex. *Structure.* 20:1757–1768. https://doi.org/10.1016/j.str.2012.07.012.

56. Mantovani, R., X. Y. Li, …, D. Mathis. 1994. Dominant-Negative Analogs of Nf-Ya. *J. Biol. Chem.* 269:20340–20346.

57. Oldfield, A. J., T. Henriques, …, R. Jothi. 2019. NF-Y controls fidelity of transcription initiation at gene promoters through maintenance of the nucleosome-depleted region. *Nat. Commun.* 10:3072. https://doi.org/10.1038/s41467-019-10905-7.

58. Slenders, E., M. Castello, …, G. Vicidomini. 2021. Confocal-based fluorescence fluctuation spectroscopy with a SPAD array detector. *Light Sci. Appl.* 10:31. https://doi.org/10.1038/s41377-021-00475-z.

Biophysical Journal, Volume *125*

Supplemental information

Spatial triple-correlation spectroscopy reveals heterotrimer dynamics in live cells

Julissa Sanchez-Velasquez, Tao Sun, Xiaomeng Zhang, and Elizabeth Hinde

Supplementary information

Spatial Triple-Correlation Spectroscopy (S3CS) Reveals Heterotrimer Dynamics in Live Cells

Julissa Sanchez-Velasquez[1], Tao Sun[1], Xiaomeng Zhang[1], Elizabeth Hinde[1*].

[1]School of Physics, University of Melbourne, Melbourne, Victoria Australia.

* Corresponding author: elizabeth.hinde@unimelb.edu.au

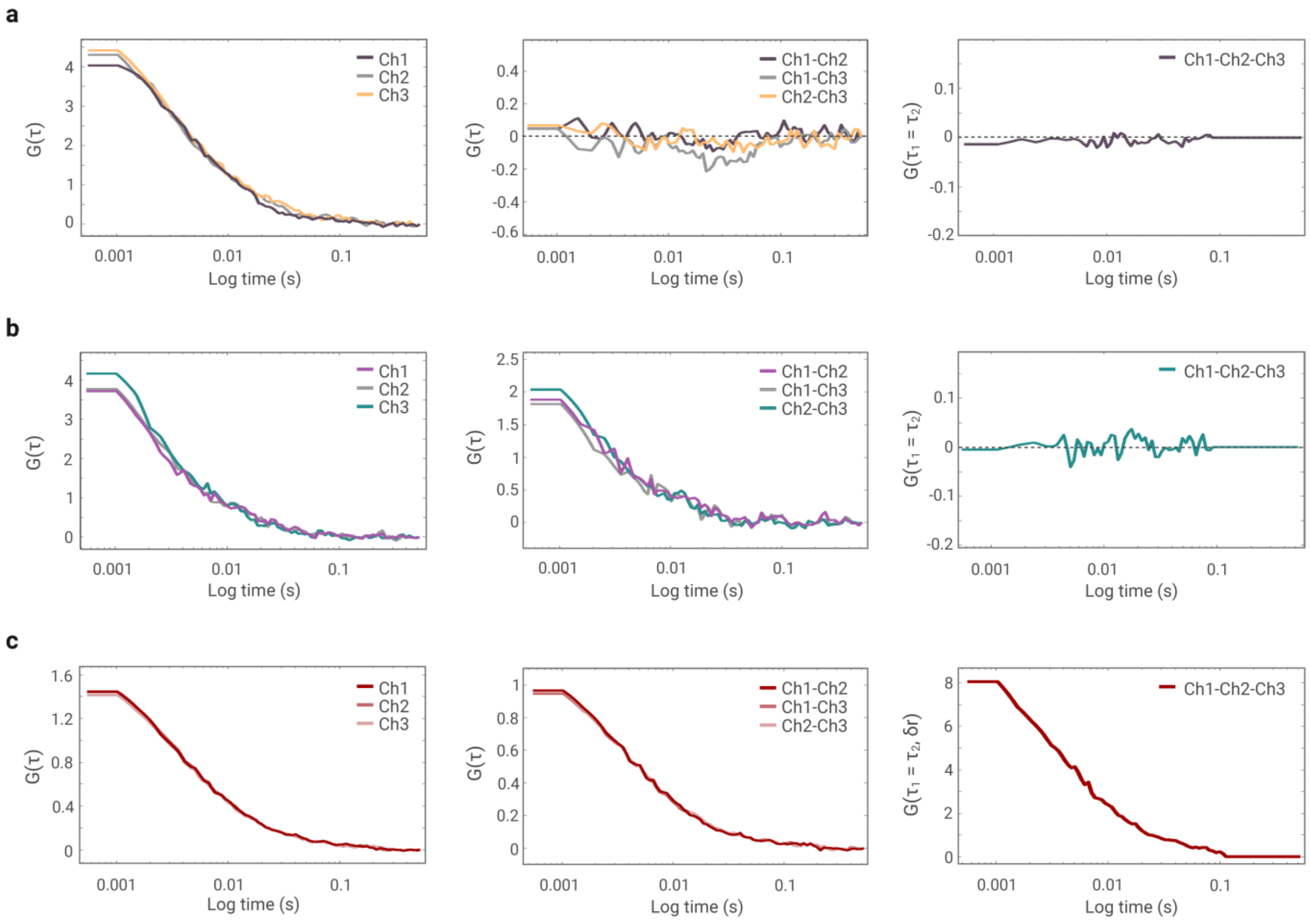

Supplementary Fig. 1. Positive S3CS triple-correlation amplitudes are specific to heterotrimeric assemblies. a-c, Representative autocorrelation (G_1, G_2, G_3) (left), cross-correlation ($G_{1\text{-}2}$, $G_{1\text{-}3}$, $G_{2\text{-}3}$) (middle), and triple correlation ($\tau_1 = \tau_2$) (right) functions obtained from simulations considering a homogeneous population of monomers (**a**), dimers (**b**), and heterotrimers (**c**) undergoing isotropic diffusion ($N = 200$, $D = 10\ \mu m^2\ s^{-1}$).

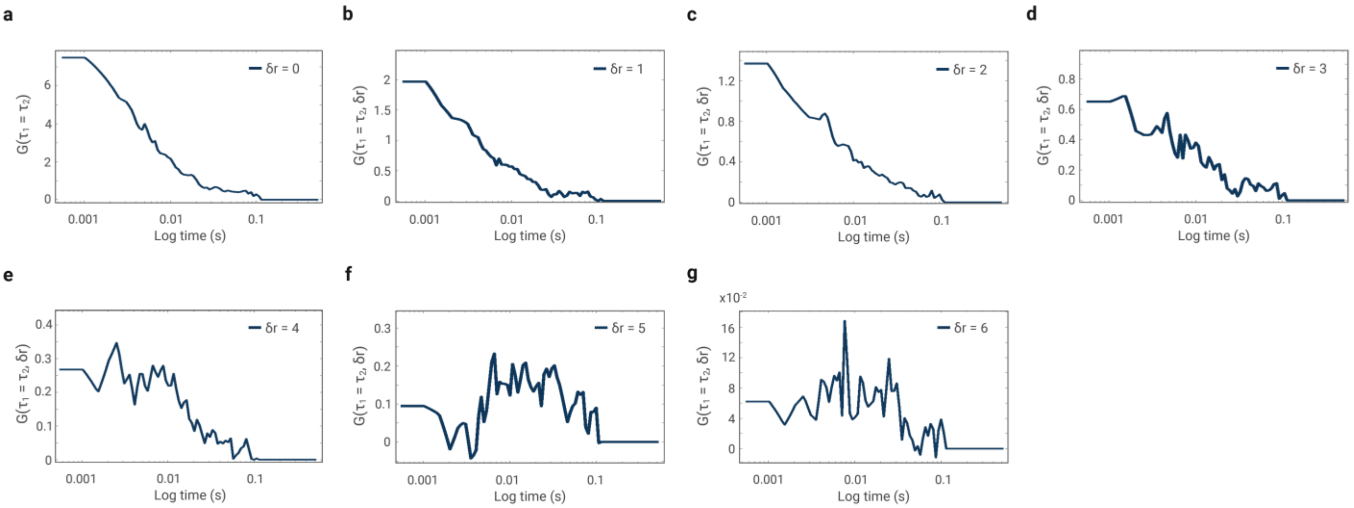

Supplementary Fig. 2. S3CS across increasing spatial offsets. a, $G_{123}(\tau_1 = \tau_2)$ profile obtained from a simulation considering a homogeneous population of heterotrimers undergoing isotropic diffusion (N = 200, D = 10 $\mu m^2\ s^{-1}$). **b-g,** $G_{123}(\tau_1 = \tau_2, \delta r)$ profiles computed at increasing spatial offsets (δr = 1, 2, 3, 4, 5, 6) from the simulation in **a**.

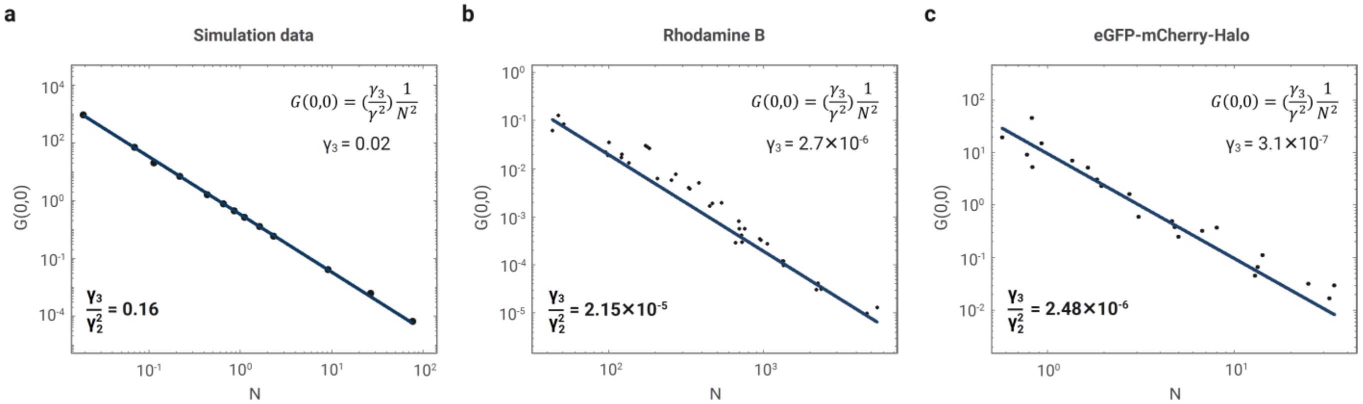

Supplementary Fig. 3. Determining the correction factor γ_3 for triple-correlation analysis. a-c, To account for the intensity distribution within the triple-correlation observation volume, triple-correlation amplitudes, $G_{123}(0,0)$, were measured across varying concentrations of simulated heterotrimers (**a**), Rhodamine B (**b**), and the eGFP-mCherry-Halo-JF646 construct (**c**). γ_3 was obtained by fitting $G_{123}(0,0)$ versus the apparent number of molecules (N) to the equation shown in the log-log plots. The resulting γ_3 values were used to calculate the ratio of triple- to double-correlation focal volumes, γ_3/γ, with γ fixed to $1/\sqrt{8}$, the value predicted for a Gaussian focal volume.

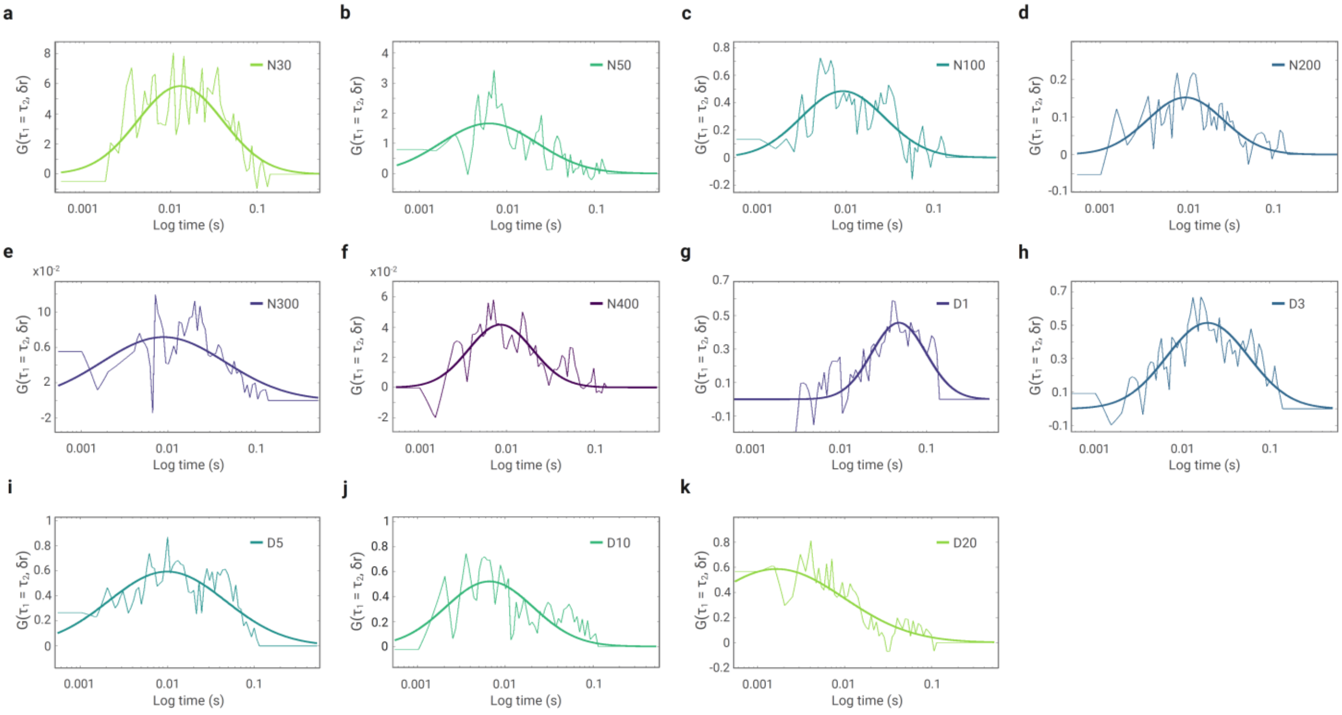

Supplementary Fig. 4. Underlying S3CS correlation profiles corresponding to Fig. 2n,p. a-f, $G_{123}(\tau_1 = \tau_2, \delta r = 6)$ profiles obtained from simulations considering a homogeneous population of heterotrimers undergoing isotropic diffusion (D = 10 μm^2 s^{-1}) at increasing particle numbers (N = 30 - 400). **g-k,** $G_{123}(\tau_1 = \tau_2, \delta r = 6)$ profiles obtained from simulations considering a homogeneous population of heterotrimers with fixed particle number (N = 100) across increasing diffusion coefficients (D = 1 – 20 μm^2 s^{-1}). Solid curves indicate fits obtained using a general Gaussian model.

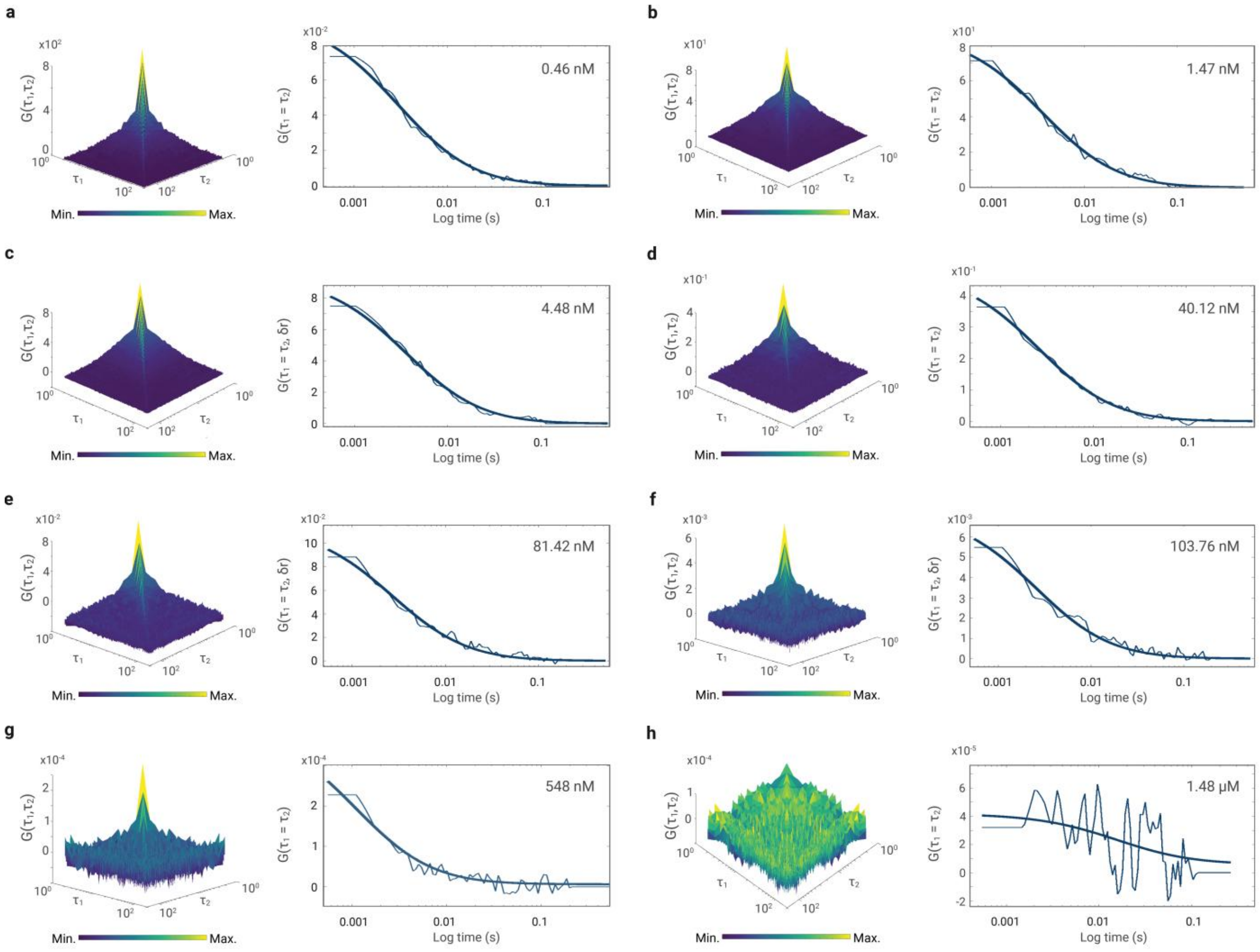

Supplementary Fig. 5. Concentration dependence of S3CS correlation profiles. a-h, Representative averaged S3CS correlation surfaces, $G_{123}(\tau_1, \tau_2)$, obtained from simulations considering a homogeneous population of heterotrimers undergoing isotropic diffusion (D = 10 μm^2 s^{-1}) across increasing particle concentrations within the observation volume (0.46 nM – 1.48 μM). For each condition, the $\tau_1 = \tau_2$ diagonal extracted from the 2D triple correlation surface is shown on the right. Solid lines indicate fits using a one-component diffusion model assuming a 3D Gaussian point spread function (PSF).

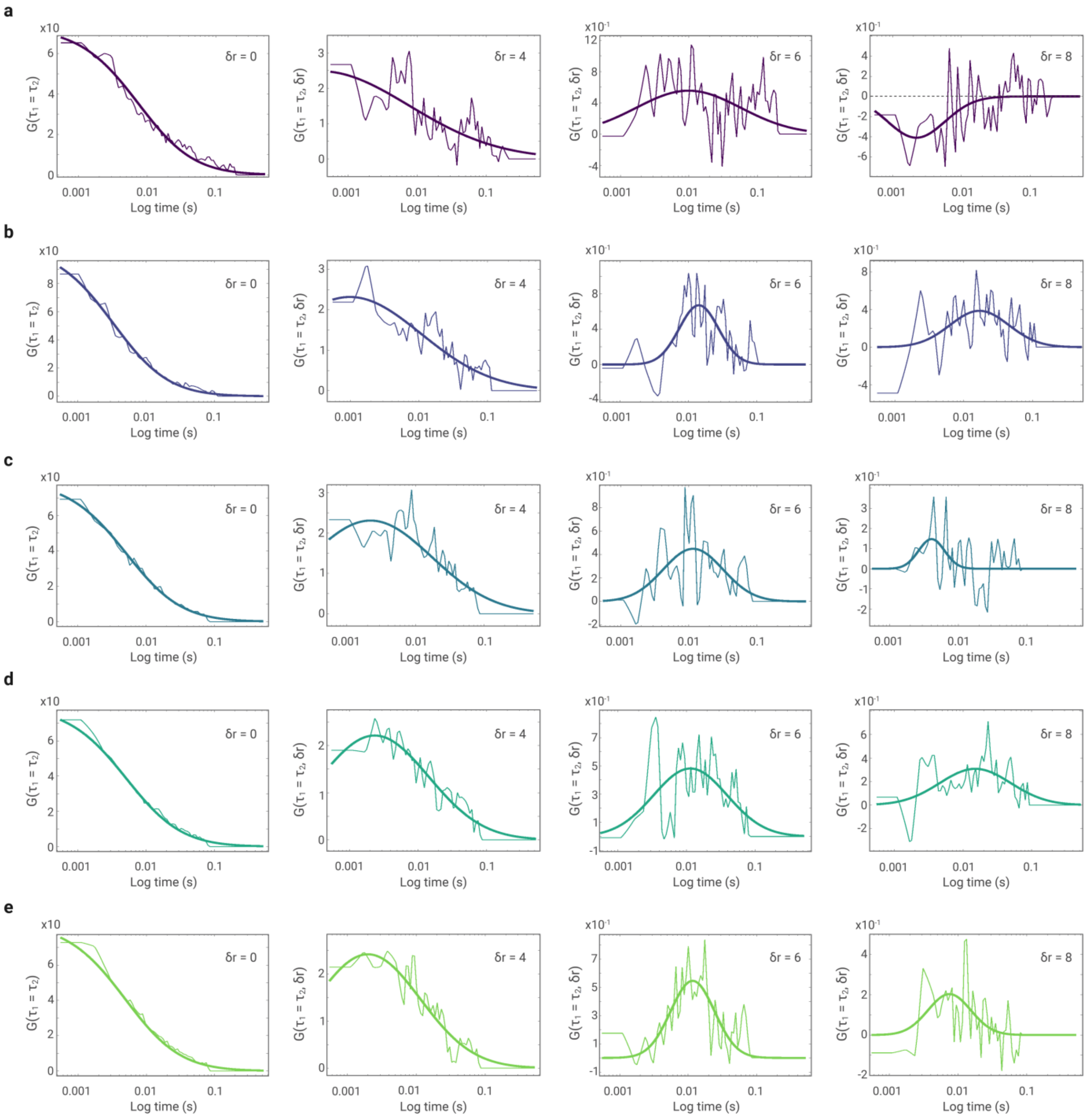

Supplementary Fig. 6. Effect of increasing statistical sampling in S3CS analysis. a-e, $G_{123}(\tau_1 = \tau_2, \delta r)$ profiles computed at increasing spatial offsets (δr = 0, 4, 6, and 8) obtained from simulations considering a homogeneous population of heterotrimers undergoing isotropic diffusion (N = 100, D = 10 $\mu m^2\ s^{-1}$). S3CS analyses were calculated using progressively increased statistical sampling, corresponding to 100 000 (**a**), 200 000 (**b**), 300 000 (**c**), 400 000 (**d**), and 500 000 (**e**) scanned lines. Solid lines indicate fits obtained using a one-component diffusion model assuming a 3D Gaussian point spread function (PSF) for δr = 0, or a general Gaussian model for δr > 0. Increasing sampling improves S3CS profiles' stability and smoothness, particularly at larger spatial offsets.

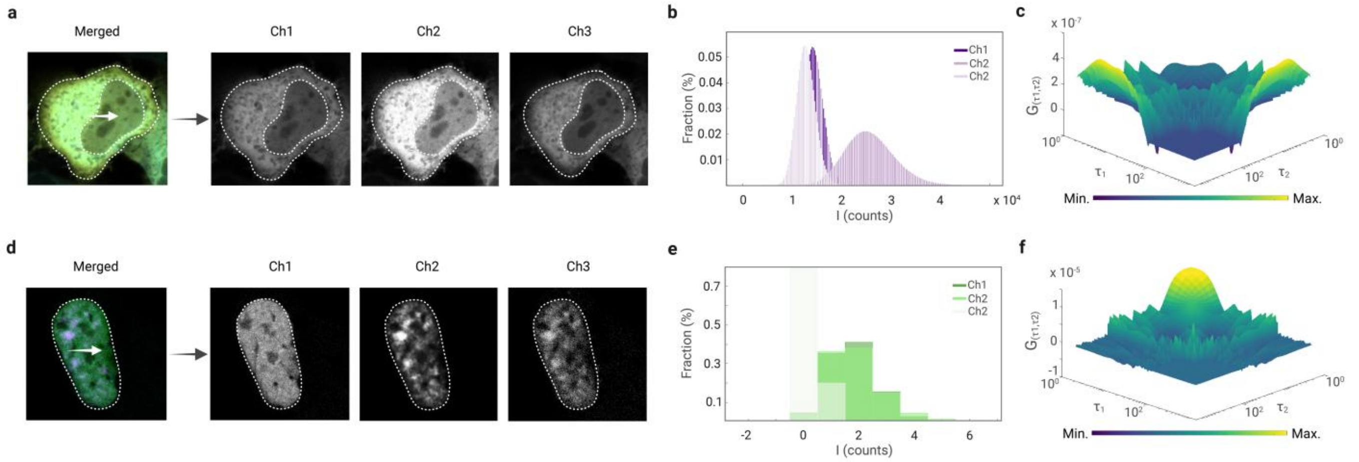

Supplementary Fig. 7. Impact of protein concentration on S3CS in live cells. a, Representative confocal image of a HeLa cell transfected with the eGFP-mCherry-Halo-JF646 construct. A three-color merged confocal image is shown on the left. Confocal images for the eGFP (Ch1), mCh (Ch2), and Halo-JF646 (Ch3) channels are shown on the right. The line across the nuclear envelope positions the directionality of the three-channel line scan. **b,** Intensity histograms for Ch1, Ch2, and Ch3 from the cell presented in **a**. **c,** $G_{123}(\tau_1, \tau_2)$ profile for the cell shown in **a**. At high protein concentration within the observation volume (~33 μM), the correlation profile exhibits increased noise. **d,** Representative confocal image of a HeLa cell co-transfected with eGFP-NF-YB, mCherry-NF-YA, and NF-YC-Halo646. A three-color merged confocal image is shown on the left. Confocal images for the eGFP (Ch1), mCh (Ch2), and Halo-JF646 (Ch3) channels are shown on the right. The line inside the nucleus positions the directionality of the three-channel line scan. **e,** Intensity histograms for Ch1, Ch2, and Ch3 from the cell presented in **d**. **f,** $G_{123}(\tau_1, \tau_2)$ profile for the cell shown in **d**. At low protein concentration (< 2 μM), a clear correlation peak is observed. I, intensity.

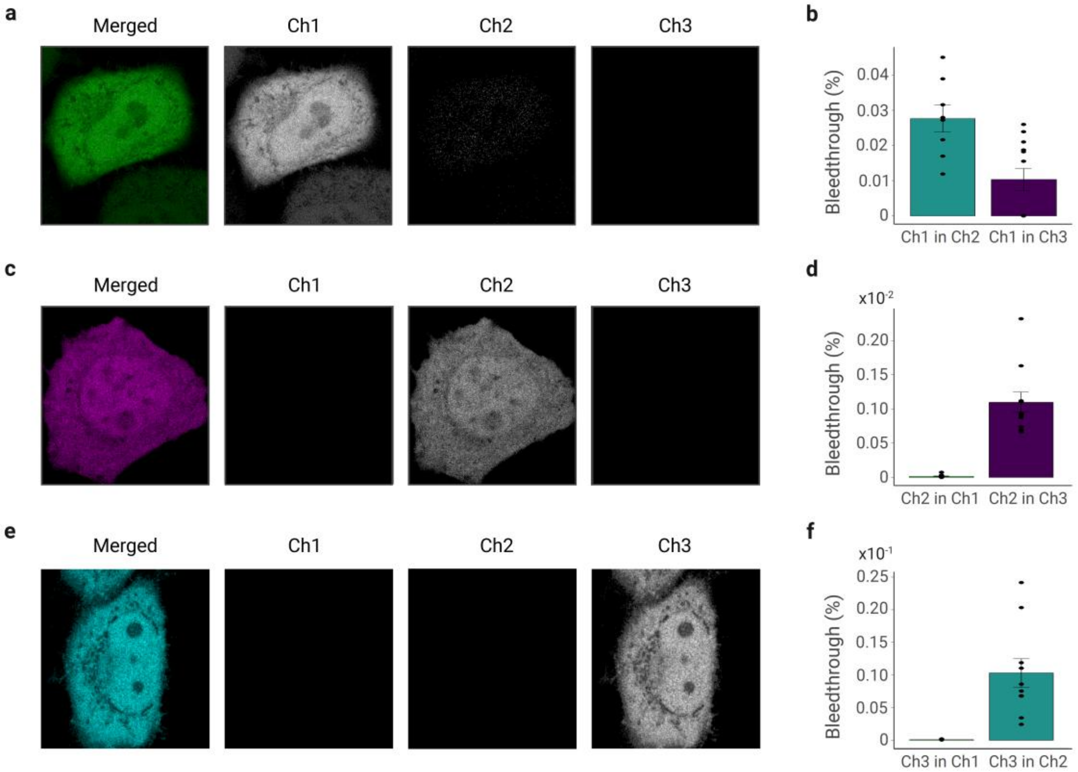

Supplementary Fig. 8. Quantitative validation of spectral bleedthrough in three-color confocal acquisitions. a, Representative confocal image of a HeLa cell expressing eGFP alone. Left: three-color merged image. Right: single-channel images for Ch1 (eGFP detection), Ch2, and Ch3. **b,** Quantification of spectral bleedthrough from eGFP into Ch2 and Ch3. For each cell, fluorescence intensity measured in Ch1 was normalized to 100%, and the corresponding signal detected in Ch2 or Ch3 was expressed as a percentage of the Ch1 intensity. **c,** Representative confocal image of a HeLa cell expressing mCherry alone, shown as a merged image (left) and individual detection channels (Ch1, Ch2, Ch3, right). **d,** Quantification of spectral bleedthrough from mCherry into Ch1 and Ch3, calculated by normalizing Ch2 (mCherry channel) intensity to 100% and expressing signals detected in the other channels as a percentage of the Ch2 intensity. **e,** Representative confocal images of a HeLa cell expressing Halo-JF646 alone, shown as merged and single-channel images. **f,** Quantification of spectral bleedthrough from Halo-JF646 into Ch1 and Ch2, with Ch3 (Halo-JF646 channel) normalized to 100% and residual signal in Ch1 and Ch2 expressed as a percentage of Ch3 intensity. In **b**,**d**,**f** bars represent mean ± s.e.m. Across all single-color controls, bleedthrough into non-corresponding detection channels was minimal, confirming effective spectral separation and validating the suitability of this imaging configuration for S3CS analyses.

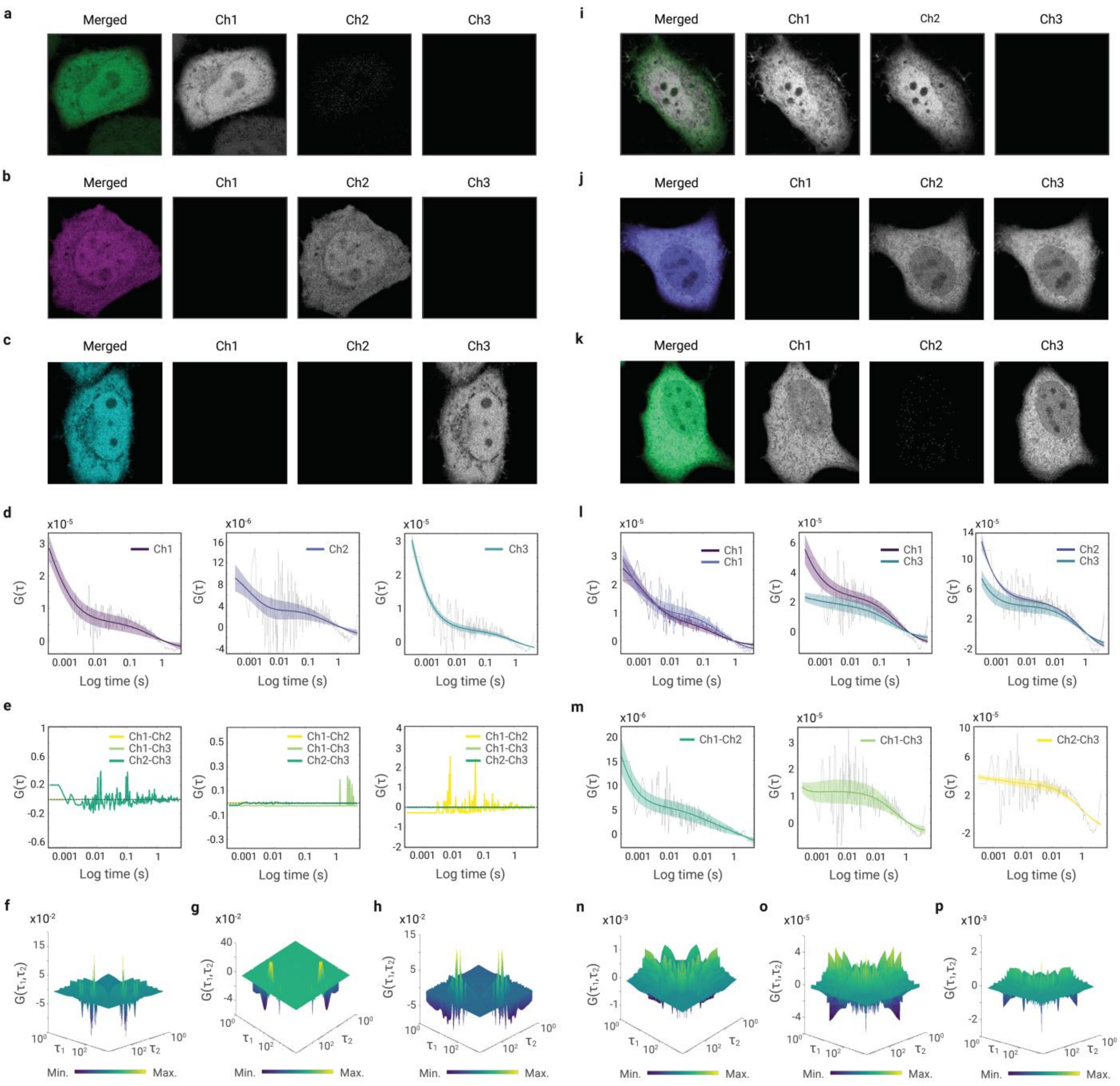

Supplementary Fig. 9. Validation of bleedthrough suppression and specificity in S3CS using independent fluorophores and dual-color constructs. a-c, Representative confocal images of HeLa cells expressing eGFP (**a**), mCherry (**b**), or Halo-JF646 (**c**). Three-color merged confocal images are shown on the left, with corresponding single-channel images for eGFP (Ch1), mCh (Ch2), and Halo-JF646 (Ch3) shown on the right. **d,** Autocorrelation functions (ACF) computed from the corresponding detection channel expressing eGFP (left), mCherry (center), and Halo-JF646 (right). **e,** Cross correlation functions (CCF) computed between all possible channel pairs for cells expressing single fluorescent proteins: eGFP (left), mCherry (center), and Halo-JF646 (right). **f-h,** Representative $G_{123}(\tau_1, \tau_2)$ profiles obtained from cells expressing eGFP (**f**), mCherry (**g**), or Halo-JF646 (**h**). **i-k,** Representative confocal images of HeLa

cells expressing dual-color fusion constructs: mCherry-eGFP (**i**), eGFP-Halo-JF646 (**j**), or mCherry-Halo-JF646 (**k**). Three-color merged images are shown on the left, with individual detection channels shown on the right. **l,** ACFs computed from the corresponding fluorescence channels for each dual-color construct: mCherry-eGFP (left), eGFP-Halo-JF646 (center), and mCherry-Halo-JF646 (right). **m,** CCFs computed between the two fluorophore channels present in each dual-color construct, mCherry-eGFP (left), eGFP-Halo-JF646 (center), and mCherry-Halo-JF646 (right). **n-p,** Representative $G_{123}(\tau_1, \tau_2)$ profiles from cells expressing mCherry-eGFP (**n**), eGFP-Halo-JF646 (**o**), and mCherry-Halo-JF646 (**p**). In all cases, triple-correlation profiles were absent, confirming negligible spectral bleedthrough and high specificity of the S3CS measurement.

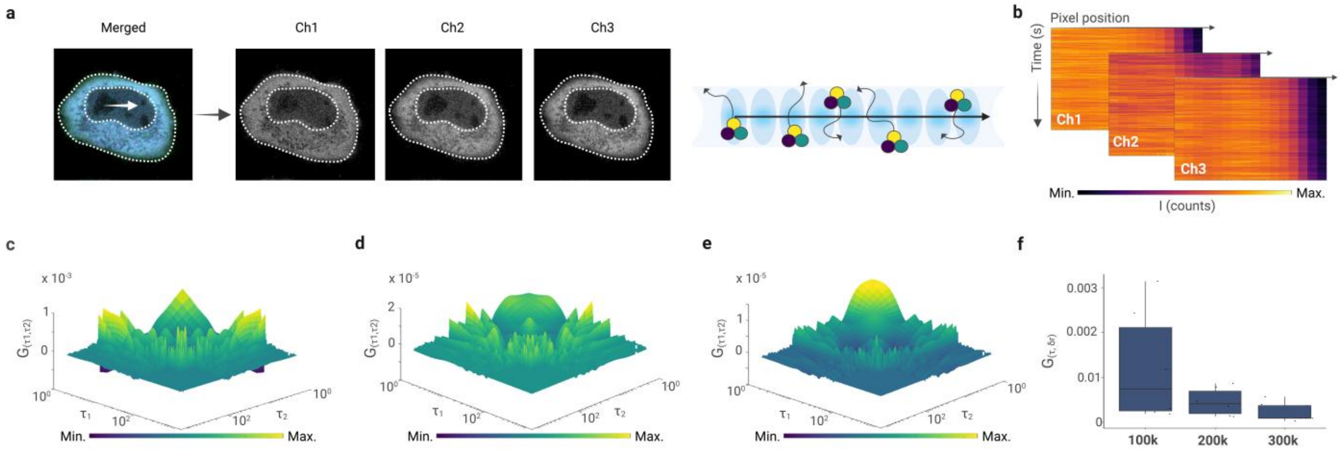

Supplementary Fig. 10. Improved definition of S3CS profiles in live cells with increased line-scan statistics. a, Representative confocal image of a HeLa cell transfected with the eGFP-mCherry-Halo-JF646 triple construct. A three-color merged confocal image is shown on the left. Confocal images for the eGFP (Ch1), mCh (Ch2), and Halo-JF646 (Ch3) channels are shown on the right. The line inside the nucleus positions the directionality of the three-channel line scan. **b,** The fluctuations in fluorescence intensity that result from eGFP-mCherry-Halo-JF646 diffusing in and out of pixels along the line scan are recorded and plotted in intensity carpet representations, where the x-axis is pixel position, and the y-axis is time. **c-e,** $G_{123}(\tau_1, \tau_2)$ profiles from line scans performed using 100 000 (**c**), 200 000 (**d**), and 300 000 (**e**) lines, demonstrating improved correlation peak definition with increased scan statistics. In **c** and **d**, the $G_{123}(\tau_1, \tau_2)$ profiles were obtained from HeLa cells transfected with the eGFP-mCherry-Halo-JF646 triple construct; in **e**, the $G_{123}(\tau_1, \tau_2)$ profile was obtained from a HeLa cell co-transfected with eGFP-NF-YB, mCherry-NF-YA, and NF-YC-Halo646. **f,** Boxplots summarizing the maximum $G_{123}(\tau_1 = \tau_2)$ amplitudes, illustrating reduced variability and improved correlation signal robustness as the number of lines increases from 100 000 (100k) to 300 000 (300k). Each box plot shows the median (middle line) and interquartile range (boxes). The bottom and top of each box indicate the 25th and 75th percentiles, respectively, while whiskers represent the minimum and maximum.

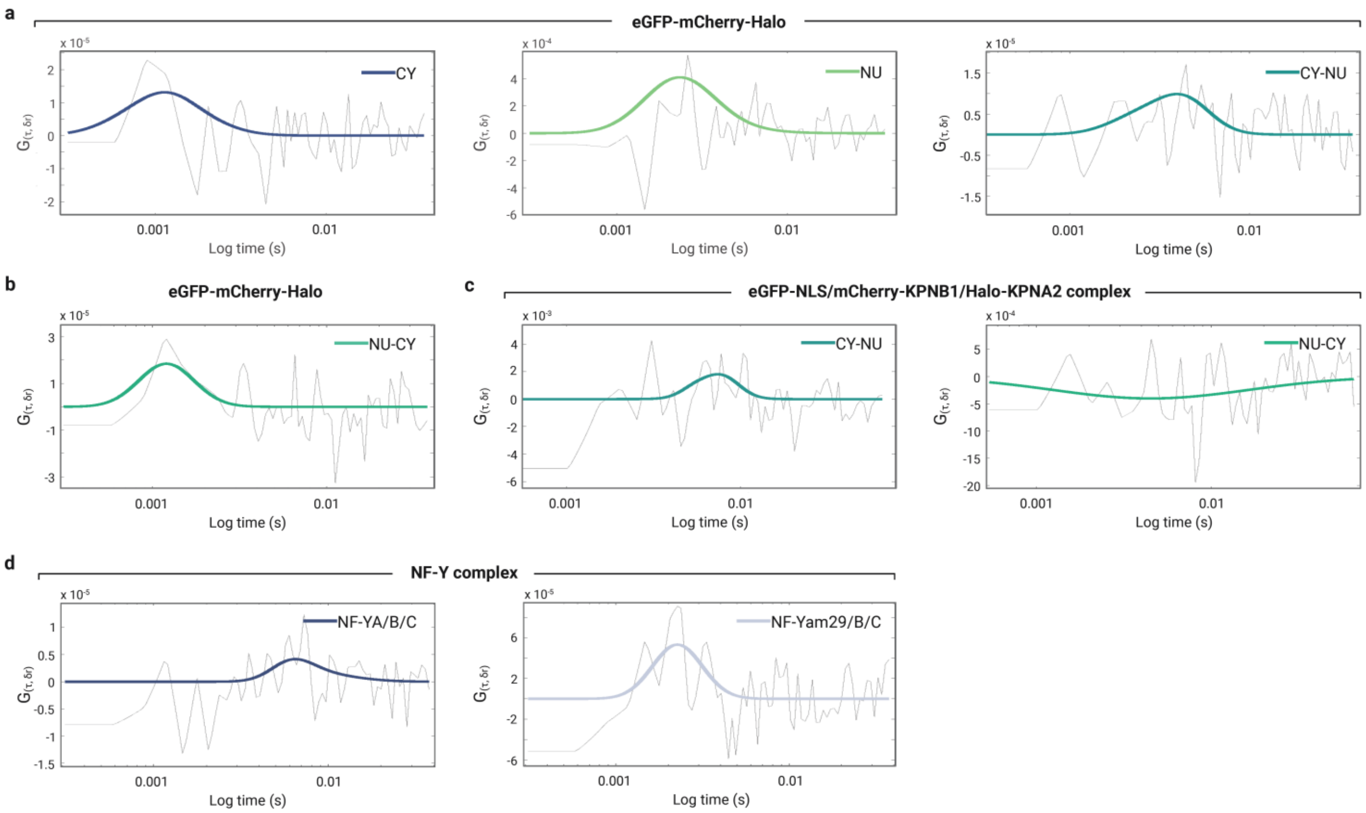

Supplementary Fig. 11. Underlying raw data for the fitted S3CS profiles shown in Figs. 4-6. a,b, Average $G_{123}(\tau_1 = \tau_2, \delta r = 3)$ profile for eGFP-mCherry-Halo-JF646 mobility within the cytoplasm (CY), nucleus (NU), and across the nuclear envelope in the cytoplasm to nucleus direction (**a**) and in the nucleus to cytoplasm direction (**b**). The solid thick lines represent the fitted curves (reference Fig. 4). **c,** Average $G_{123}(\tau_1 = \tau_2, \delta r = 3)$ profile for eGFP-NLS/mCherry-KPNB1/Halo-JF646-KPNA2 complex mobility across the nuclear envelope in the cytoplasm to nucleus direction (left) and in the nucleus to cytoplasm direction (right). The solid thick lines represent the fitted curves (reference Fig. 5). **d,** Average $G_{123}(\tau_1 = \tau_2, \delta r = 3)$ profiles for NF-YA/B/C (left) and NF-Yam29/B/C (right) complexes mobility inside the nucleus. The solid thick lines represent the fitted curves (reference Fig. 6).

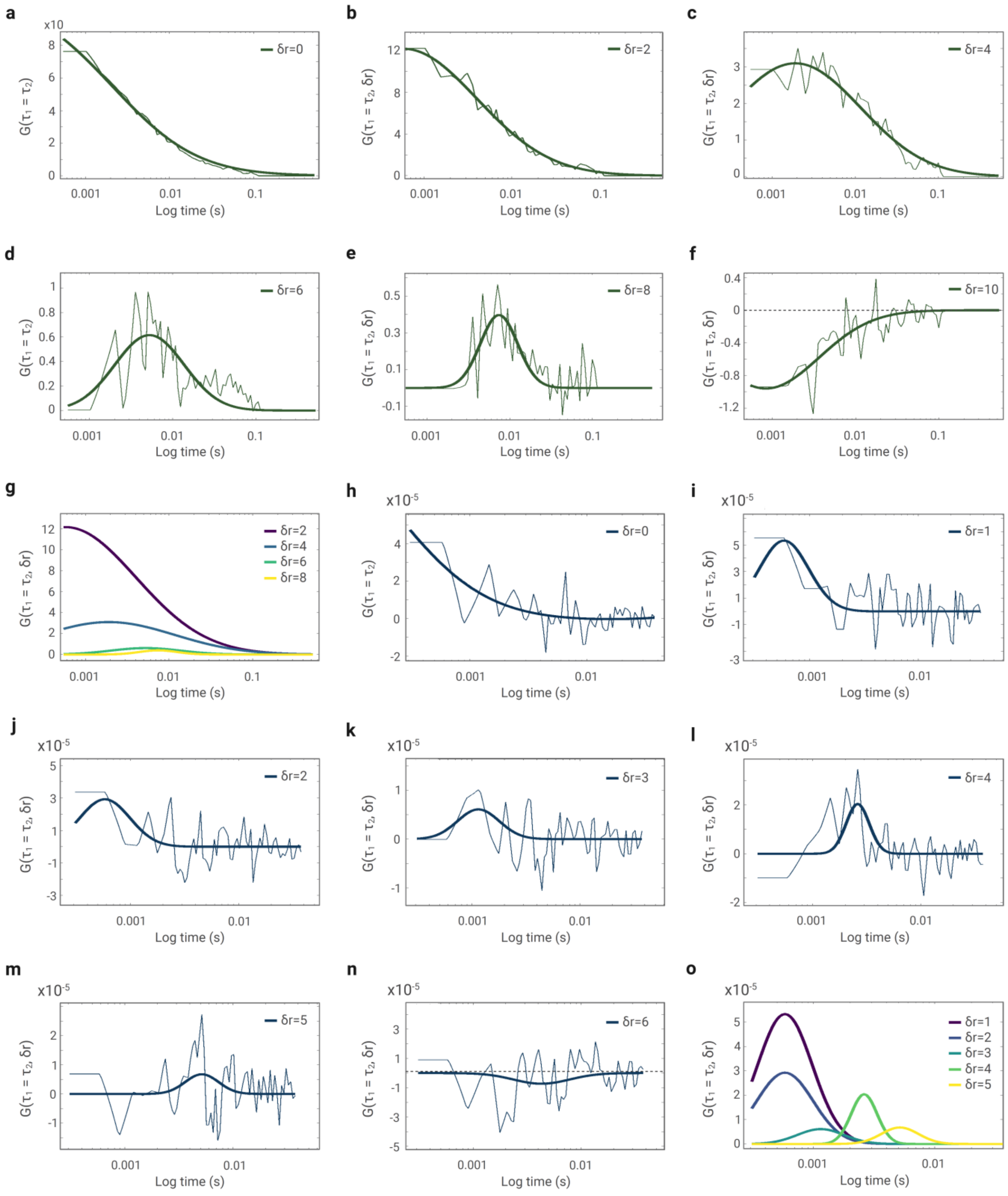

Supplementary Fig. 12. Spatial range of detectable S3CS correlations in simulations and live cells. **a,** Schematic of S3CS analysis, $G_{123}(\tau_1 = \tau_2)$, from simulations considering a homogeneous population of heterotrimers undergoing isotropic diffusion (N = 100, D = 10 μm^2 s^{-1}). **b-f,** Simulated $G_{123}(\tau_1 = \tau_2, \delta r)$

profiles computed at increasing spatial offsets (δr = 2, 4, 6, 8, 10) from the simulation in **a**, showing well-defined correlation profiles at small-to-intermediate offsets, with progressive signal attenuation and loss of detectable correlation at the largest offset ($\delta r = 10$). **g,** Fitted $G_{123}(\tau_1 = \tau_2, \delta r)$ profiles corresponding to panels **b-e**. **h,** Experimental $G_{123}(\tau_1 = \tau_2)$ measured in live HeLa cells expressing the eGFP-mCherry-Halo646 triple-labelled construct. **i-n,** Experimental $G_{123}(\tau_1 = \tau_2, \delta r)$ profiles computed at increasing spatial offsets (δr = 1, 2, 3, 4, 5, 6), showing a progressive reduction in correlation amplitude and increased noise at larger offsets. **o,** Fitted $G_{123}(\tau_1 = \tau_2, \delta r)$ profiles corresponding to panels **i-m**. Solid lines indicate fits obtained using a one-component diffusion model assuming a 3D Gaussian point spread function (PSF) for $\delta r = 0$, or a general Gaussian model for $\delta r > 0$.
